# Combined spatially resolved metabolomics and spatial transcriptomics reveal the mechanism of RACK1‐mediated fatty acid synthesis

**DOI:** 10.1002/1878-0261.13752

**Published:** 2024-10-18

**Authors:** Lixiu Xu, Jinqiu Li, Junqi Ma, Ayshamgul Hasim

**Affiliations:** ^1^ Department of Basic Medicine Xinjiang Medical University and Xinjiang Key Laboratory of Molecular Biology of Endemic Diseases Urumqi China; ^2^ Department of Pathology QiLu Hospital of Shandong University (Qingdao) China; ^3^ Department of Gynecology The First Affiliated Hospital of Xinjiang Medical University Urumqi China

**Keywords:** cervical cancer, metabolic alterations, RACK1, spatial transcriptomics, spatially resolved metabolomics

## Abstract

Lipid metabolism is altered in rapidly proliferating cancer cells, where fatty acids (FAs) are utilized in the synthesis of sphingolipids and glycerophospholipids to produce cell membranes and signaling molecules. Receptor for activated C‐kinase 1 (RACK1; also known as small ribosomal subunit protein) is an intracellular scaffolding protein involved in signaling pathways. Whether such lipid metabolism is regulated by RACK1 is unknown. Here, integrated spatially resolved metabolomics and spatial transcriptomics revealed that accumulation of lipids in cervical cancer (CC) samples correlated with overexpression of *RACK1*, and RACK1 promoted lipid synthesis in CC cells. Chromatin immunoprecipitation verified binding of sterol regulatory element‐binding protein 1 (SREBP1) to acetyl‐CoA carboxylase (ACC) and fatty acid synthase (FASN) promoters. RACK1 enhanced *de novo* FA synthesis by upregulating expression of sterol regulatory element binding transcription factor 1 (*SREBP1*) and lipogenic genes *FASN* and *ACC1*. Co‐immunoprecipitation and western blotting revealed that RACK1 interacted with protein kinase B (AKT) to activate the AKT/mammalian target of rapamycin (mTOR)/SREBP1 signaling pathway to promote FA synthesis. Cell proliferation and apoptosis experiments suggested that RACK1‐regulated FA synthesis is key in the progression of CC. Thus, RACK1 enhanced lipid synthesis through the AKT/mTOR/SREBP1 signaling pathway to promote the growth of CC cells. RACK1 may become a therapeutic target for CC.

## Graphical abstract

The authors analyzed the spatial distributions of gene and metabolite profiles in cervical cancer through spatial transcriptomic and spatially resolved metabolomic techniques. Pivotal genes and metabolites within these cases were then identified and validated. These findings unveil the role of RACK1 in AKT/mTOR/SREBP1‐signaling‐mediated fatty acid synthesis. RACK1 therefore contributes to the intricate process of cervical carcinogenesis.

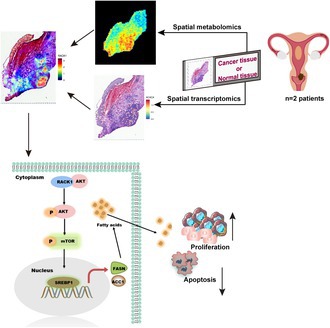

**Abbreviations**
ACACA: acetyl‐CoA carboxylase
ACC: acetyl‐CoA carboxylase
AKT: protein kinase B
CC: cervical cancer
DMEM: Dulbecco's minimum essential medium
FASN: fatty acid synthase
KEGG: Kyoto Encyclopedia of Genes and Genomes
MSI: mass spectrometry imaging
mTOR: mammalian target of rapamycin
RACK1: receptor for activated C‐kinase 1
SEM: standard error of the mean
SM: spatially resolved metabolomics
SREBP1: sterol regulatory element binding protein 1
ST: spatial transcriptomics

## 1 Introduction

Cervical cancer is the malignant tumor of the female reproductive system with the highest incidence worldwide, and more than 600 000 new cases are diagnosed annually with the number of deaths reaching nearly 340 000 every year [1]. Malignant proliferation and lymph node metastasis are the primary reasons for the poor prognosis of cervical cancer, and the 5‐year survival rate decreases from 93% to 42% after lymph node metastasis occurs [2]. However, no effective method to prevent or control malignant proliferation and lymph node metastasis exists. Therefore, identification of novel, promising targets as a therapeutic intervention for patients with cervical cancer is a key goal for research.

The reprogramming of cellular energy metabolism is now recognized as a hallmark of cancer, and an increasing number of studies have reported that lipid metabolic reprogramming provides conditions for rapid proliferation of tumor cells [3]. In our previous study, plasma and tissue samples from patients with cervical cancer were subjected to metabonomic analyses by HRMAS‐^1^HNMR spectroscopy, and UHPLC–MS/MS suggested that distinct metabonomic signatures could distinguish among CC; in particular, abnormal fatty acid metabolism is closely related to malignant proliferation and lymph node metastasis of cervical cancer [4, 5]. Although the metabolomes have improved our understanding of cancer, the distribution of the discriminating metabolites and gene expression across the cancer remains poorly understood.

The RACK1 is a highly conserved scaffolding protein containing a tryptophan‐aspartic acid domain (WD) repeat structure. This special structure of RACK1 molecular surface determines its intracellular function, participates in the interaction of various proteins, regulates the activities of kinases, phosphodiesterase and phosphatases, subcellular localisation, and substrate specificity, and affects many signal molecules and signal transduction pathways, as well as gene expression [6, 7]. RACK1 interacts with the apoptotic protein Fem1b to promote its ubiquitination level in colon cancer cells; downregulation of RACK1 leads to FEM1B‐mediated apoptosis [8]. RACK1 enhances the M2/M1 macrophage ratio and promotes OSCC cell proliferation through the NF‐KB pathway [9]. As a prognostic factor of lung squamous cell carcinoma, RACK1 is involved in glycolysis and promotes cell proliferation [10]. We previously identified the upregulation of RACK1 in cervical cancer. Moreover, RACK1 participates in glycolysis of CC [11]. The above results suggest that RACK1 plays an important role in tumor cell metabolism and proliferation. However, the molecular mechanism of regulation of fatty acid metabolism by RACK1 remains poorly understood.

Therefore, we employed a strategy of integrating spatial metabolomics with spatial transcriptomics analysis to screen discriminating metabolites (primarily enrichment in lipids) between cancerous and normal tissues and explore the relationship between lipid metabolism and RACK1. This study provided a novel approach to gain further insight into CC pathophysiology.

## 2 Materials and methods

### 2.1 Tissue sample collection

The study has been approved by the Ethics Committee of the First Affiliated Hospital of the Xinjiang Medical University (authorization no. IACUC‐20220304‐01). Signed informed consent was obtained from patients in the study, and all the research was performed in accordance with the Declaration of Helsinki and according to national and international guidelines. The CC patients were pathologically diagnosed without chemo/radiotherapy before surgical operation between May 2020 and September 2022. The embedded samples were stored at −80 °C before being sectioned.

### 2.2 Spatial metabolomic technology and spatial transcriptomic sequencing

#### 2.2.1 Spatial Metabolomic technology

##### 2.2.1.1 Chemicals and reagents

Acetonitrile of MS‐grade was sourced from Thermo Fisher Scientific (Waltham, MA, USA). Purified water was obtained from Watsons (Hongkong, China), and formic acid was supplied by Merk (Sigma‐Aldrich Chemie GmbH, Taufkirchen, Germany). The tissue freezing medium was purchased from Leica (Leica Microsystems, Wetzlar, Germany), while Eosin Y‐solution (0.5% aqueous) and hematoxylin were obtained from Sigma‐Aldrich (St. Louis, MO, USA).

##### 2.2.1.2 Sample preparation

Tissue samples were cut into approximately 10 sagittal slices, each 10 μm thick, using a cryostat microtome (Leica CM 1950, Leica Microsystems) and mounted on positively charged desorption plates (Thermo Scientific). The sections were stored at −80 °C before analysis. Prior to MSI analysis, the samples were dried at −20 °C for 1 h and then equilibrated at room temperature for 2 h. Another adjacent slice was used for H&E staining.

##### 2.2.1.3 Data acquisition and MSI analysis

MSI was performed using an AFADESI‐MSI platform (Beijing Victor Technology Co., LTD, Beijing, China) connected to a Q‐Orbitrap mass spectrometer (Q Exactive, Thermo Scientific). The solvent used was a mixture of ACN/H_2_O (8 : 2) in negative mode and ACN/H_2_O (8 : 2, 0.1% FA) in positive mode, with a flow rate of 5 μL·min^−1^. Transport gas flow was maintained at 45 L·min^−1^, with a spray voltage of 7 kV, and a sample‐to‐sprayer distance of 3 mm (the same distance as between the sprayer and the ion transport tube). MS resolution was set at 70 000, the mass detection range was 70–1000 Da, the automated gain control (AGC) target was 2E6, and the maximum injection time was set at 200 ms. The S‐lens voltage was maintained at 55 V, and the capillary temperature was 350 °C. The MSI experiments involved scanning the tissue surface at a constant speed of 0.2 mm·s^−1^ along the *x*‐axis, with a 100 μm step in the *y*‐axis.

##### 2.2.1.4 Data processing

The .raw files collected were converted into .imML format and imported into MSiReader (an open‐source tool for analyzing high‐resolution MS imaging data on the Matlab platform). After background subtraction, ion images were reconstructed, and region‐specific MS profiles were accurately extracted by aligning them with high‐resolution H&E images. To identify distinguishing endogenous molecules from different tissue microregions, OPLS‐DA (Orthogonal Partial Least Squares Discriminant Analysis) was applied. Variable Importance in Projection (VIP) scores from the OPLS‐DA model were used to rank each variable's contribution to group differentiation. A two‐tailed Student's *t*‐test was employed to assess the statistical significance of differences in metabolites between groups, with differential metabolites selected based on VIP values greater than 1.0 and *P*‐values below 0.05.

##### 2.2.1.5 Analyte identification

For metabolite annotation, a method controlling the false discovery rate (FDR) was applied, which ensured the identification of metabolites in high‐resolution imaging mass spectrometry data. The identified metabolites were then subjected to high‐resolution tandem mass spectrometry directly from the tissue sections.

#### 2.2.2 Spatial transcriptomic sequencing

##### 2.2.2.1 Cryosectioning, H&E Staining and bright‐field imaging for ST

Tissues were obtained under the supervision of senior pathologists, with 4–5 mm^3^ pieces containing IMPC components being selected. These pieces were embedded in O.C.T. Compound (Torrance, CA, USA) and frozen in isopentane pre‐chilled to −80 °C. Tissue sections, 10 μm thick, were cut using a −20 °C cryostat microtome (Leica, CM1950) and mounted onto gene expression slides. The sections were then stained with H&E, and bright‐field images were captured at 10× magnification using a 3D HISTECH Pannoramic MIDI FL whole‐slide scanner.

##### 2.2.2.2 ST barcoded microarray information

The spatial transcriptomics (ST) microarray used for library preparation in this study (Visium Gene Expression Slide) was obtained from 10× Genomics (https://www.10xgenomics.com/). Each spot on the slide has a diameter of 55 μm with a center‐to‐center distance of 100 μm, covering an area of 6.5 mm by 6.5 mm. Each slide includes four capture areas (each 6.5 × 6.5 mm), with 5000 spatially barcoded spots per capture area.

##### 2.2.2.3 Tissue optimization

Cryosections, 10 μm thick, were placed on the six capture areas of the slide, while the remaining two areas were used as positive and negative controls. The six cryosections were exposed to varying permeabilization times to capture mRNA, followed by reverse transcription to produce fluorescently labeled cDNA. The optimal permeabilization time was determined based on the highest fluorescence signal with minimal diffusion.

##### 2.2.2.4 Permeabilization, cDNA amplification, library construction, and sequencing

The mRNA released from cells in each spot was captured by poly(dT) sequences, followed by the addition of Reverse Transcription Master Mix to initiate cDNA synthesis with spatial barcode information. Second Strand Mix was subsequently added for second‐strand cDNA synthesis. The cDNA was transferred from the slide for amplification and library construction. cDNA fragments (200–300 bp) were generated by chemical fragmentation, end‐repaired, poly(A)‐tailed, and selected. After library quality control, paired‐end 150 bp (PE150) sequencing was performed using the NovaSeq 6000 platform.

##### 2.2.2.5 ST data analysis

The space ranger software (Shanghai Luming biological technology co., LTD, Shanghai, China) was employed to count effective barcodes and UMIs, generating a gene‐barcode expression matrix for each sample, including barcode‐labeled spots and gene expression counts. The spots and gene expression were evaluated through visualized distributions of gene numbers and UMI counts. UMAP (Uniform Manifold Approximation and Projection) was applied for dimensionality reduction.

### 2.3 Cell lines and cell culture

Human cervical cancer cells SiHa (RRID:CVCL_0032) and C‐33a (RRID:CVCL_1094) were purchased from Pricella Life Scinence &Technology Co., Ltd (Wuhan, China). They were cultured in Dulbecco's minimum essential medium (DMEM) containing 10% (vol/vol) fetal bovine serum (FBS; Gibco, Grand Island, NY, USA) and 100 units·mL^−1^ penicillin–streptomycin. All cell lines were authenticated using STR profiling to confirm their identities. Cells were cultured at 37 °C with 5% CO_2_ and routinely passaged at 90% intensity. All cell lines were identified and certified by Pricella Life Scinence &Technology Co., Ltd and routinely checked for mycoplasma contamination to ensure the integrity and reliability of the cell lines.

### 2.4 Lentivirus‐mediated stable low or high expression cells

Lentiviruses of RACK1‐knockdown (shRACK1), SREBP1‐overexpression (OE SREBP1) and negative control were purchased from GeneChem (Shanghai, China). The sequences of shRACK1 were as follows: shRACK1‐1, 5′‐AGCTGAAGACCAACCACAT‐3′; shRACK1‐2, 5′‐TGTGGTTATCTCCTCAGAT‐3′; the sequences of OE SREBP1, 5′‐CCACTCCATTGAAGATGTA‐3′; the sequences of negative control, 5′‐TTCTCCGAACGTGTCACGT‐3′. Supernatant was collected after culturing for 48 h. For CC cell infection, cells (1 × 10^4^ cell per well) were cultured for 24 h, and recombinant lentivirus in serum‐free growth medium was then added at a multiplicity of infection (MOI = 30) at 37 °C for 72 h. Stable cell lines were selected using 5 μg·mL^−1^ puromycin (Sigma‐Aldrich Chemie GmbH, Taufkirchen, Germany) and the knockdown efficiency was confirmed using western blot analysis.

### 2.5 Western blot

Total protein was isolated from CC cells using radioimmunoprecipitation assay (RIPA) lysis buffer (P0013, Beyotime, Beijing, China). The protein concentration was determined with bicinchoninic acid protein assay kit (Bio‐Rad, Hercules, CA, USA). The protein was quantified to 30 μg by adding the loading buffer and phosphate‐buffered saline (PBS) in proportion. Then proteins were subjected to 10% sodium dodecyl sulfate (SDS)‐polyacrylamide gel electrophoresis and transferred onto polyvinylidene fluoride membranes. The membranes were blocked with 5% nonfat milk and probed with specific antibodies against RACK1, FASN, AKT, p‐AKT, mTOR, p‐mTOR, β‐actin, SREBP1, and ACC1 overnight at 4 °C. After washing three times in PBS containing 0.1% Tween 20, the membranes were probed with anti‐mouse or anti‐rabbit immunoglobulin coupled to horseradish peroxidase for 2 h at room temperature. The signals were visualized with an enhanced chemiluminescence detection kit (Amersham Biosciences, Little Chalfont, UK). The gray value of the target protein was imaged and used to calculate target protein expression. The primary and secondary antibodies used in this study are listed in Table S1.

### 2.6 BODIPY 493/503

Cellular neutral lipids were stained with the fluorescence dye BODIPY 493/503 (Invitrogen, Thermo Fisher Scientific Inc., USA) according to the manufacturer's instructions. 10^5^ CC cells were seeded into 10‐cm dishes, fixed with 4% paraformaldehyde for 30 min, then stained with BODIPY 493/503 (1 μg·mL^−1^) for 30 min. The dye was aspirated, and cells were washed three times with PBS for 3 min each time. Then DAPI was added and incubated in the dark for 5–10 min at room temperature. Images were acquired with an Olympus FV‐1000 confocal microscope (Olympus, Tokyo, Japan), and the staining intensities of neutral lipids were quantified using the imagej software (National Institutes of Health, Bethesda, MD, USA).

### 2.7 Colony formation assay

According to the manufacturer's instructions, for colony formation assays, 100 CC cells were seeded into six‐well plates and cultured for 1~2 weeks. Colonies were fixed in 4% paraformaldehyde for 30 min and stained with 0.5% crystal violet. The number of colonies formed in each plate was manually counted using imagej.

### 2.8 Flow cytometry

BD Annexin V‐Enhanced Green Fluorescent Protein (EGFP) Kits (BD Biosciences, Franklin Lakes, NJ, USA) were used to detect apoptosis. After transfection for 48 h, the cells were collected and washed with PBS three times. The cells were mixed with 100 μL binding buffer, and the cell suspension was adjusted to 1 × 10^6^·mL^−1^. Annexin V‐fluorescein isothiocyanate (5 μL) and phycoerythrin (5 μL) were added and incubated for 15 min. The apoptosis rate of cells in each group was immediately detected by flow cytometry.

Cells (1 × 10^6^ cells per well) were harvested by centrifugation and washed thrice with precooled PBS. The supernatant was removed and 70% ethanol precooled at −20 °C was gradually added overnight. The cells were centrifuged at 350 ***g*** for 3 min, the supernatant was removed, washed twice with 2 mL of PBS, the supernatant was centrifuged, and resuspended in 500 μL of PBS. The cells were stained with 50 μg·mL^−1^ propidium iodide (PI) solution and 100 μg·mL^−1^ RNase A (C1052, Beyotime Biotechnology Co., LTD, Shanghai, China). Finally, the cell cycle distribution of each group was promptly detected by flow cytometry.

### 2.9 Chromatin immunoprecipitation (ChIP)

ChIP assay was carried out using Chromatin Immunoprecipitation Kits (Cell Signaling Technology, Danvers, MA, USA). Cells were cross‐linked with 37% formaldehyde for 15 min, next, the chromatin was randomly broken by ultrasonication for 10 s for a total of 15 times at intervals of 10 s. The chromatin fragments were centrifuged at 13 000 ***g*** at 4 °C, and the supernatant was collected and incubated overnight at 4 °C with anti‐SREBP1 antibody and normal IgG (negative control), then digested with proteinase K. After phenol–chloroform extraction and ethanol precipitation of the resulting DNA, PCR was performed to amplify the ACC1 and FASN promoters. Precipitated DNA was analyzed by PCR; promoter primer information for ACC1 and FASN is listed in Table S2. Amplification products were then examined by electrophoresis.

### 2.10 Co‐immunoprecipitation (Co‐IP)

One microgram of anti‐RACK1 or anti‐AKT or anti‐IgG (negative control) antibodies was added separately to approximately 3 × 10^6^ CC cell lysates and incubated at 4 °C overnight. 200 μL RIPA lysis buffer was added to 10 μL of protein A/G magnetic beads (Bimake Biotechnology Co., Ltd. Houston, TX, USA) and centrifuged (4 °C, 2392 ***g***) three times for 3 min each. The pretreated beads were added to the CC cell lysate, incubated with antibodies at 4°C overnight, and centrifuged (4°C, 2392 ***g***) for 3 min. The supernatant was discarded and washed three times with wash buffer for 60 s each, followed by centrifugation (4°C, 2392 ***g***) for 30 s. The supernatant was discarded and the magnetic beads retained, 80 μL washing buffer then 20 μL of 4 × SDS loading buffer were added. The sample was then boiled at 100 °C for 5 min, placed on a magnetic rack for magnetic separation, and collected for western blot analysis.

### 2.11 Treatment of cells with chemicals

Diluted oleic acid (HY‐N1446, MedChemExpress, Shanghai, China) was added to CC cells using OA concentrations of 0, 25，50，75，100，125，150，175，and 200 μm and incubated for 24 h for detection of CC cell activity. To potentiate AKT‐induced fatty acid synthesis, cells were pretreated with the AKT activator SC79 (HY‐18749, MedChemExpress, Shanghai, China) at 10 μm for 24 h at 37 °C. To alleviate SREBP1‐induced fatty acid synthesis, 5 × 10^5^ cells per well were treated with SC79 (10 μm for 24 h) and the SREBP1 inhibitor, Fatostatin (HY‐14452, MedChemExpress), at 3 μm for 24 h at 37 °C. Reagents trans‐C75 (HY‐12364A) and PF‐05175157 (HY‐12942) were purchased from MedChemExpress. 5 × 10^5^ cells per well were treated with 50 μm trans‐C75 or 25 μm PF‐05175157 for 24 h at 37 °C. Finally, the cells were collected for western blot and proliferation‐ and apoptosis‐related experiments.

### 2.12 CCK‐8 assay

CC cell viability was determined by the Cell Counting Kit‐8 (CCK‐8) assay according to the manufacturer's instructions (PF00004, Proteintech Group Biotechnology Co. LTD., Wuhan, China). In brief, 2 × 10^3^ cells per well were inoculated into 96‐well plates (CCP‐96H, Sevier Biotechnology Co. LTD, Wuhan, China). After grown for 24, 36, and 48h, 10 μL CCK‐8 and 90 μL medium reagent were added to each well and incubated for 1.5 h at 37 °C in the dark. Finally, the absorption values at 450 nm were measured with a Multiskan FC microplatereader (1410101, Thermo Fisher Scientific Inc.) to determine the relative cellular proliferation capacities.

### 2.13 Determination of cellular free fatty acids (FFA)

The levels of cellular long‐chain free FFAs were quantified using the Free Fatty Acid Assay Kit from Abcam (Cambridge, MA, USA, Cat#ab65341). Briefly, 1 × 106 cells were homogenized in 200 μL of 1% Triton X‐100 in chloroform. The organic phase was then separated by centrifugation and air‐dried at 50 °C for 60 min. The dried lipids were reconstituted in Fatty Acid Assay Buffer and converted into acyl‐CoA by incubating with 2 μL of Acyl‐CoA Synthetase (ACS) Reagent for 30 min at 37 °C in a black 96‐well plate. Subsequently, 50 μL of reaction buffer was added to each well to initiate the oxidation of acyl‐CoA. The fluorescence intensity of the resulting product was measured using a Spark™10M multimode microplate reader with excitation/emission wavelengths set at Ex/Em = 535/587 nm.

### 2.14 Statistical analysis

The results are presented as the mean ± standard error of the mean (SEM). IBM spss Statistics for Windows, version 17.0 (IBM Corporation, Armonk, NY, USA) and graphpad prism software (5.0 version; GraphPad Software, La Jolla, CA, USA) were used for the statistical analyses. A two‐tailed unpaired Student's *t*‐test and one‐way analysis of variance (ANOVA) with Tukey's *post hoc* test were used for comparisons between two or multiple groups, respectively. Correlations between measured variables were analyzed using Spearman's rank correlation analysis. A value of *P* < 0.05 was considered to indicate a statistically significant difference.

## 3 Results

### 3.1 Region‐specific metabolic molecule profiling in cervical cancer

**Fig. 1:**
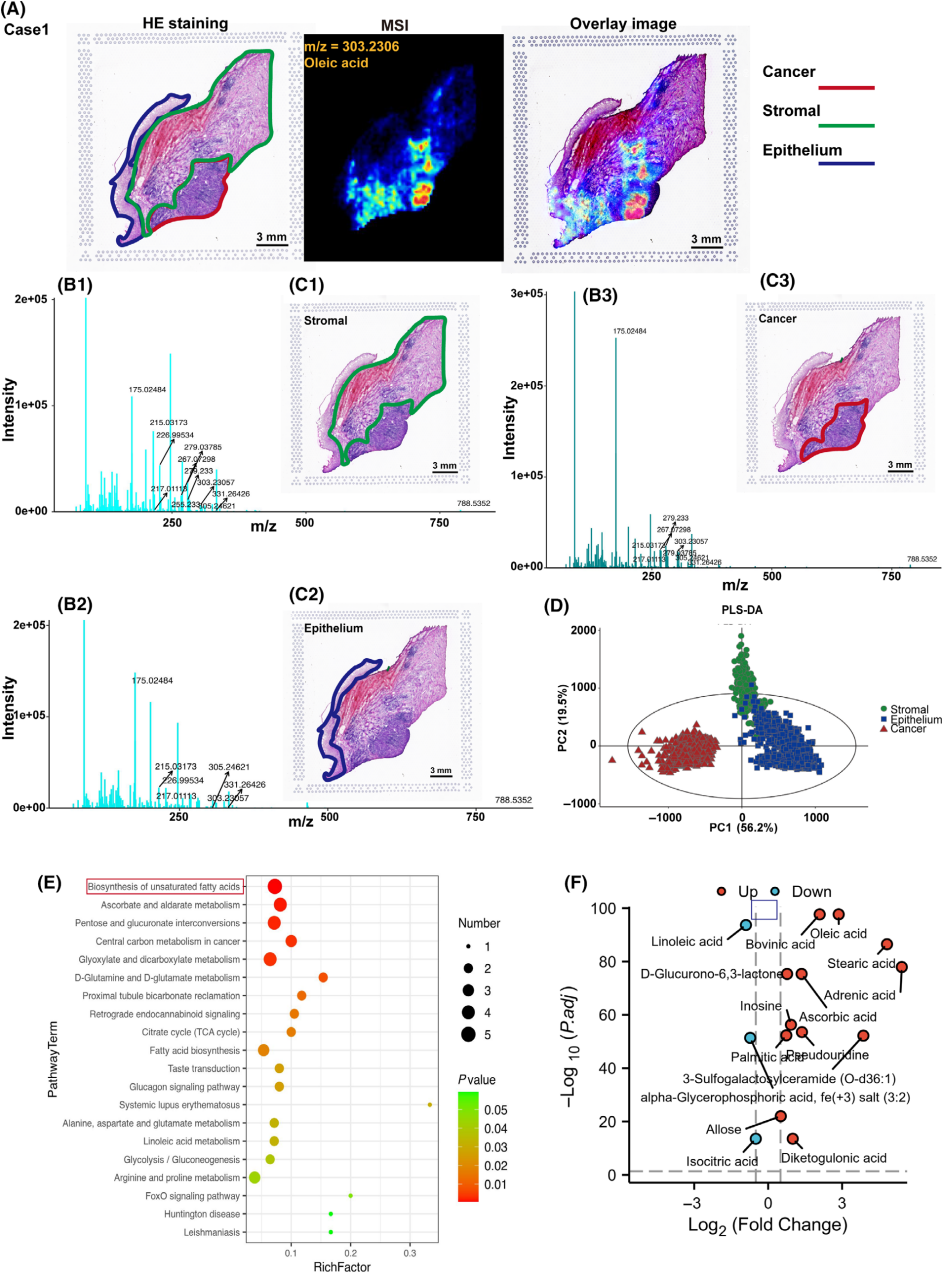
The strategy to extract region‐specific MS spectra in heterogeneous CC tissue. (A) H&E‐stained tissue images of case 1 with marked cancer (red), normal (blue), and stromal (green) tissue regions (left), the Mass Spectrometry Imaging (MSI) of case 1 (middle), the microscopy‐MSI overlay of case 1 (right). (B) Representative mass spectra for stromal tissue (B1), normal epithelium tissue (B2) and cancerous tissue (B3). (C1–C3) Digitized image of the corresponding H&E stained section for this sample. (D) OPLS‐DA score plots for cancer tissue (red triangle), stromal tissue (green dot) and epithelial tissue (blue square) of case1. (E) Metabolic pathway analysis. (F) The volcano plot displays the results of the UP/DOWN regulated significant metabolites. Scale bars, 3000 μm. CC, cervical cancer; H&E, Hematoxylin Eosin; MS, Mass Spectrometry; MSI, Mass Spectrometry Imaging; OPLS‐DA, Orthogonal Partial Least Squares Discriminant Analysis.

We identified two distinct histological categories of CC tissue: malignant tissue and epithelial tissue. Following tissue MSI analysis, microscopy images were combined with MS images to generate a microscopy‐MSI overlay. This overlay provides both the high spatial resolution of microscopy and the molecular signatures captured by MSI in a unified image (Fig. 1A and Fig. S1A). Using these overlay images, we were able to accurately extract mass spectra specific to cancer and epithelial tissues, as shown in Fig. 1B,C and Fig. S1C,D. The 15 key differential metabolites between cancer and epithelial tissues are clearly presented in these figures as well as in Table 1. To identify global molecular differences between tissue types, a partial least squares discriminant analysis (PLS‐DA) model was developed using MS image pixel data to identify tissue‐specific biomarkers. As illustrated in Fig. 1D and Fig. S1B, the PLS‐DA models based on (±) AFADESI‐MSI data effectively distinguished cancer, epithelial, and stromal tissues. Initial screening of region‐specific metabolite biomarkers was performed using classification loadings, followed by independent *t*‐tests to confirm the significance of the metabolites distinguishing cancerous from normal tissues.

**Table 1:** Identification of differential metabolites.

Metabolites	Molecular formula	Observed m/z	Related pathway	Adj.*P*‐value
Bovinic acid	C_18_H_32_O_2_	279.23300	Linoleic acid metabolism	2.08586E‐98
Oleic acid	C_18_H_34_O_2_	303.23057	Biosynthesis of unsaturated fatty acids|Fatty acid biosynthesis	2.08586E‐98
Linoleic acid	C_18_H_32_O_2_	279.23300	Biosynthesis of unsaturated fatty acids|Linoleic acid metabolism	2.06E‐94
Stearic acid	C_18_H_36_O_2_	305.24621	Biosynthesis of unsaturated fatty acids|Fatty acid biosynthesis	3.29745E‐87
Adrenic acid	C_22_H_36_O_2_	331.26426	Biosynthesis of unsaturated fatty acids	1.2003E‐78
D‐Glucurono‐6,3‐lactone	C_6_H_8_O_6_	175.02484	Ascorbate and aldarate metabolism	4.53306E‐76
Ascorbic acid	C_6_H_8_O_6_	175.02484	Ascorbate and aldarate metabolism	4.53306E‐76
Inosine	C_10_H_12_N_4_O_5_	267.07298	Purine metabolism	5.19364E‐57
Pseudouridine	C_9_H_12_N_2_O_6_	279.03785	Pyrimidine metabolism	2.96839E‐54
Palmitic acid	C_16_H_32_O_2_	255.23300	Biosynthesis of unsaturated fatty acids|Fatty acid biosynthesis|Fatty acid elongation|Fatty acid degradation	4.85287E‐53
3‐Sulfogalactosylceramide (O‐d36:1)	C_42_H_81_NO_11_S	788.53520	Sphingolipid metabolism	6.59106E‐53
alpha‐Glycerophosphoric acid, fe(+3) salt (3:2)	C_3_H_9_O_6_P	217.01113	ABC transporters|Glycerophospholipid metabolism|Choline metabolism in cancer|Glycerolipid metabolism	4.36193E‐52
Allose	C_6_H_12_O_6_	215.03173	ABC transporters|Fructose and mannose metabolism	9.97928E‐23
Isocitric acid	C_6_H_8_O_7_	226.99534	Central carbon metabolism in cancer|Glyoxylate and dicarboxylate metabolism|Citrate cycle (TCA cycle)|Glucagon signaling pathway	2.98289E‐14
Diketogulonic acid	C_6_H_8_O_7_	226.99534	Ascorbate and aldarate metabolism|Pentose and glucuronate interconversions	2.98289E‐14

Among the significantly changed metabolites of Cases 1 and 2, the Kyoto Encyclopedia of Genes and Genomes (KEGG, www.kegg.jp) dataset suggested that unsaturated fatty acid metabolic pathways were significantly deregulated in CC (Fig. 1E and Fig. S1E). Then, combined with the result of KEGG and Volcano Plot, oleic acid was identified as a candidate metabolite among the differential metabolites (Fig. 1F). Based on the IC50 values of oleic acid (Fig. S1F), 0, 10, and 25 μm were selected for subsequent experiments. Next, the CCK8 and colony formation suggested the oleic acid enhanced the proliferation of CC cells (Fig. S1G,H). These data suggested that FA accumulation in tumor regions contributes to the development and progression of CC.

### 3.2 Visualization of fatty acid synthesis‐related spatial genes expression

**Fig. 2:**
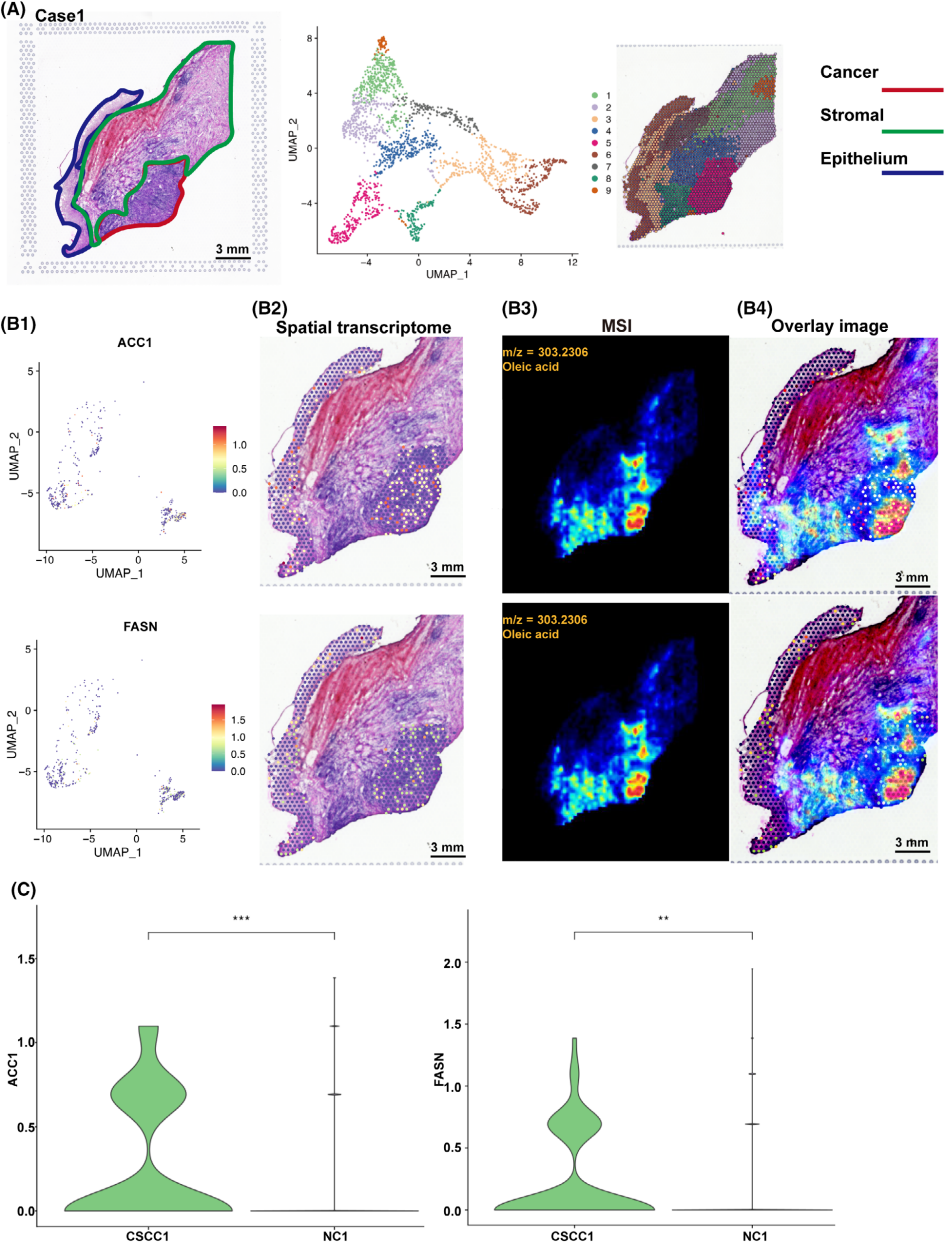
Tumor morphology and hierarchical clustering results. (A) H&E‐stained tissue images of case 1 with marked cancer (red), normal (blue), and stromal (green) tissue regions (left). UMAP plot performed on the gene expression data from spots covered by tissue (middle). UMAP clustering has identified nine groups of spots, which have been assigned a color each (right). (B1) UMAP plot of case1 performed on the *ACC1* (top) and *FASN* (bottom) expression data from spots covered by tissue. (B2) The spatial distributions of *ACC1* gene (top) and *FASN* gene (bottom). (B3) MS images and levels of oleic acid. (B4) The overlay of *ACC1* spatial expression and oleic acid (up), *FASN* spatial expression and oleic acid (bottom). (C) Violin Plot of *ACC1* (left) and *FASN* (right) gene expression across 2 clusters with 561 spots in CC (cluster 5 with 261 spots (cancer) and cluster 6 with 301 spots (normal)). Data were represented as mean ± standard deviation and compared with the NC, (**P < 0.01 or ***P < 0.001, independent sample *t*‐tests were used for comparisons between two groups). Scale bars, 3000 μm. ACC1, acetyl‐CoA carboxylase 1; CC, cervical cancer; FASN, fatty acid Synthase; H&E, Hematoxylin Eosin; MS, Mass Spectrometry; NC, normal control; UMAP, Uniform Manifold Approximation and Projection.

We acquired transcriptome data for a total of 8872 spots from the two patients. In addition, 17 157 UMIs (median) and 3505 genes (median) per spot were ascertained (Table S3). Next, we demultiplexed the reads and identified their spatial locations in these tissue samples using location‐specific barcodes (Fig. 2A and Fig. S2A). We investigated special location of RNA transcripts corresponding to enzymes involved in lipids metabolism. ST analyses revealed RNA transcript levels of acetyl‐CoA carboxylase (*ACC1*, which synthesizes malonyl‐CoA from acetyl‐CoA) and fatty acid synthase (*FASN*, which catalyzes the endogenous FAs to synthesize long‐chain FAs), to be highly enriched in the tumor region from Case 1, while no significant difference was observed in Case 2 (Fig. 2B,C and Fig. S2B,C). These two results exhibit inconsistency, potentially attributable to individual variations or insufficient sample size. These data collectively support higher activity of the FA synthases in the cancer region. To this end, direct comparison of spatial MALDI‐MS and ST data from the same region demonstrated that mRNA levels of *ACC1* and *FASN* in CC region strongly coincided with enrichment of oleic acid (Fig. 2B and Fig. S2B).

### 3.3 RACK1 significantly improved the lipid content and expression levels of fatty acid synthesis enzymes in CC cells

**Fig. 3:**
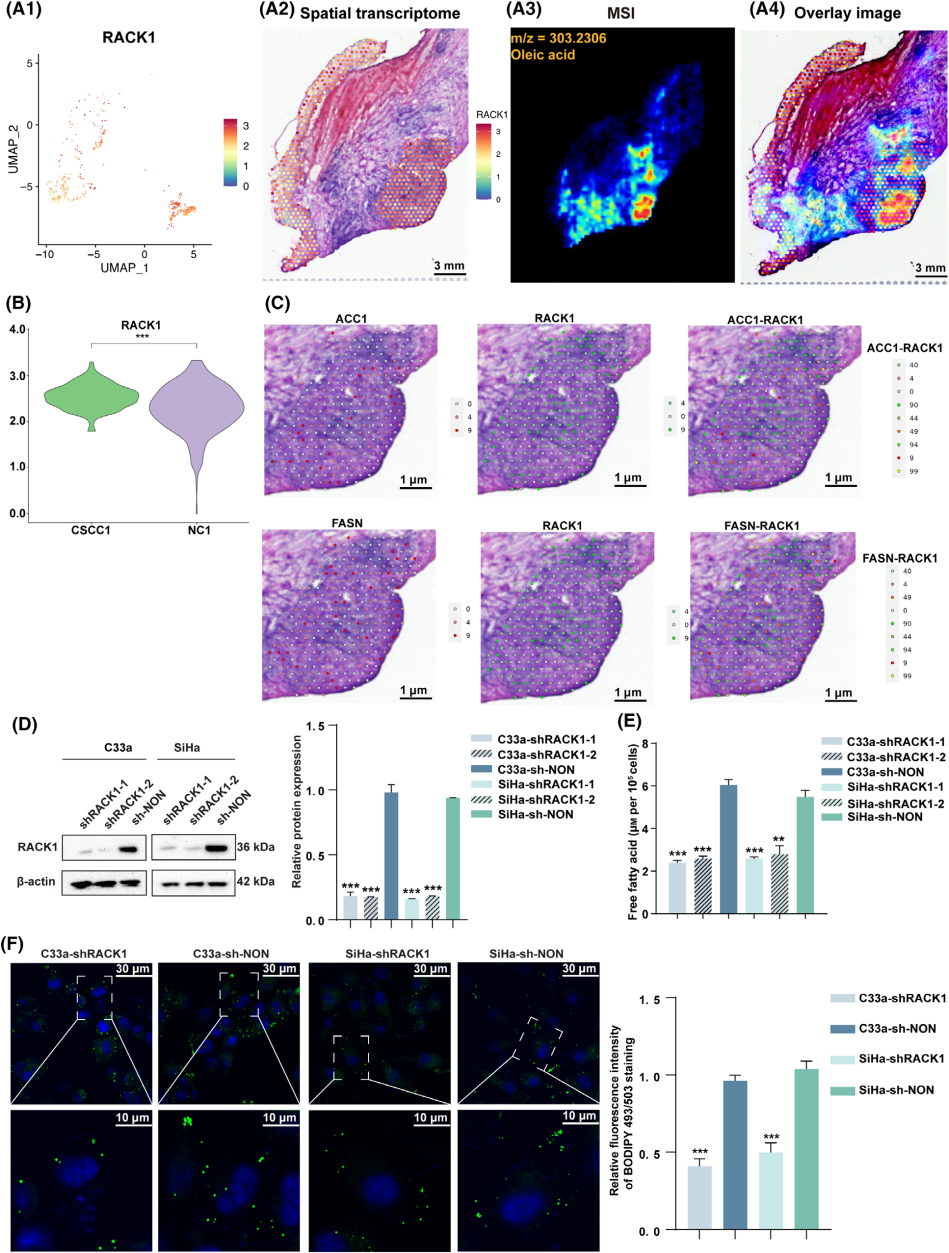
RACK1 significantly improved lipid contents and expression levels of fatty acid in CC cells. (A1) UMAP plot performed on the *RACK1* expression data from spots covered by tissue of case 1. (A2) The spatial distributions of *RACK1* gene. (A3) MS images and levels of oleic acid. (A4) The overlay of *RACK1* spatial expression and oleic acid. (B) Violin Plot of *RACK1* gene expression across 2 clusters with 561 spots in CC (cluster 5 with 261 spots (cancer) and cluster 6 with 301 spots (normal)). (C) Spatial transcriptomics images demonstrated enrichment of *ACC1*, *RACK1*, and *FASN* transcript levels to be co‐localized in the cancer region of case 1. The spatial distributions of *ACC1* (top left), *RACK1* (middle), and *FASN* (bottom left), the overlay of *ACC1* spatial expression and *RACK1* spatial expression (top right), and the overlay of *FASN* spatial expression and *RACK1* spatial expression (bottom right). (D) RACK1 knockdown in CC cells was verified by western blot analysis (left), with the quantified bands assessed (right, *n* = 3). (E) Intracellular free fatty acid levels in CC cell with RACK1 knockdown (*n* = 3). (F) Neutral lipid content detection by staining with fluorescence dye BODIPY 493/503 in CC cells with RACK1 knockdown (left), with the quantified bands assessed (right, *n* = 3). Data were compared with NC (***P < 0.001) or sh‐NON, (**P < 0.01 or ***P < 0.001, independent sample *t*‐tests were used for comparisons between two groups, the mean ± standard deviation of experiments conducted in triplicate, using one‐way ANOVA). Scale bars, 1 or 10 or 30 or 3000 μm. The average fluorescence intensity per cell was analyzed using imagej (*n* = 30 cells per group). ACC1, acetyl‐CoA carboxylase 1; CC, cervical cancer; FASN, fatty acid Synthase; MS, mass spectrometry; RACK1, receptor for activated C‐kinase 1; UMAP, uniform manifold approximation and projection.

To further evaluate the relationship between FA metabolism and *RACK1* expression of CC, we performed MALDI‐MS and ST imaging of tissue sections. The findings revealed a significant overlap in the spatial distributions of oleic acid and *RACK1* mRNA (Fig. 3A and Fig. S3A), with a high level of *RACK1* observed in the CC region (Fig. 3B and Fig. S3B). Furthermore, the mRNA level of *RACK1* was in accordance with *ACC1* and *FASN* in the CC region (Fig. 3C and Fig. S3C). Previous studies verified RACK1 overexpression in CC samples and cell [11]. To further examine the role of RACK1 in fatty acid anabolism, changes in free fatty acid and lipid content in CC cells after RACK1 knockdown were preliminarily detected. Stable downexpression of RACK1 in CC cells was verified by measuring protein expression levels through western blotting (Fig. 3D). RACK1 knockdown significantly decreased intracellular free fatty acid levels (Fig. 3E). Consistently, the fluorescent lipophilic dye BODIPY 493/503 also showed that the intracellular lipid content was significantly reduced with RACK1 downregulation (Fig. 3F). The reduction in cellular lipid content may be attributed to the inhibition of lipid biosynthesis, decreased uptake of fatty acids, and impaired fatty acid oxidation. Therefore, the expression levels of key molecules involved in fatty acid synthesis (ACC1, FASN), fatty acid uptake (CD36), and fatty acid oxidation (CPT1A) were assessed in CC cells following RACK1 knockdown. Compared with the shNON group, protein expression of the key enzymes involved in fatty acid synthesis (ACC1 and FASN) decreased after RACK1 knockdown, while the levels of key factors involved in fatty acid uptake and oxidation remained unchanged (Fig. S3D,E). The above results indicate that changes in RACK1 can affect fatty acid synthesis, while they have no effect on fatty acid uptake and oxidation. For further support, qRT‐PCR was used to detect the expression of RACK1, ACC1, and FASN in 25 CC tissue samples. Spearman rank correlation analysis showed significant positive correlations between the expression of *RACK1* and *ACC1* (*r* = 0.816, *P* < 0.001) and *FASN* (*r* = 0.722, *P* < 0.001) (Fig. S3F).

### 3.4 RACK1 increased fatty acid synthesis enzyme expression by activating AKT/mTOR signaling

**Fig. 4:**
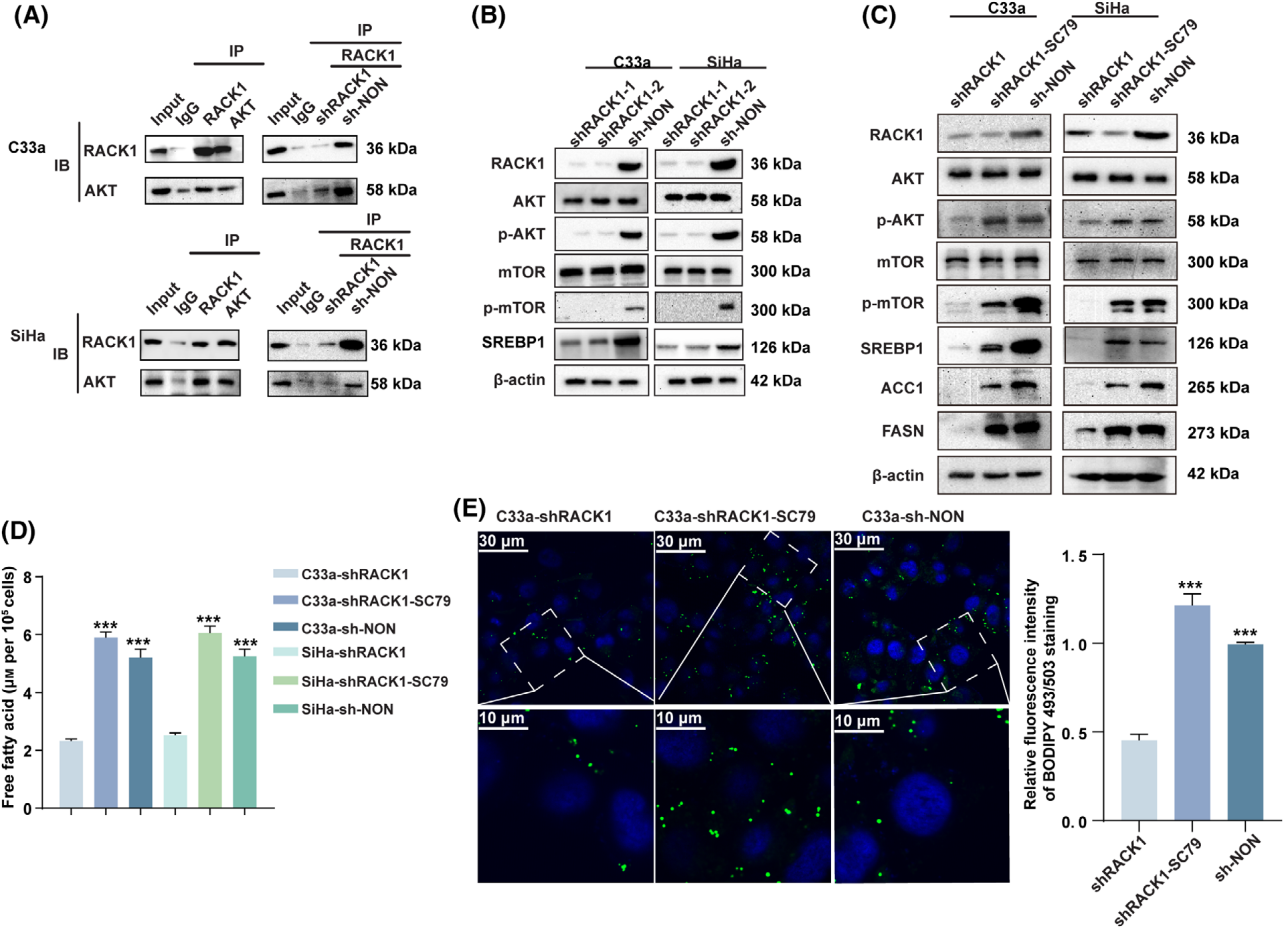
RACK1 improved the lipid content levels of CC cells by activating AKT/mTOR signaling. (A) The Co‐IP and western blot results validated the effects of RACK1 binding to AKT in CC cells. (B) Western blot analysis for protein expression levels of p‐AKT (ser472 + S474 + S473), AKT, p‐mTOR (S2448), total mTOR and SREBP1 in CC cell with RACK1 knockdown. shRACK1 cells was stimulated with SC79. (C) The RACK1, p‐AKT (ser472 + ser474 + ser473), p‐mTOR (ser2448), AKT, mTOR, SREBP1, ACC1 and FASN expression were examined using western blot analysis. (D) Intracellular free fatty acid levels. (E) Neutral lipid content detection by staining with fluorescence dye BODIPY 493/503 in C33a cell (left), with the quantified bands assessed. Shown is one representative of three independent experiments. Data were compared with the shRACK1 group (***P < 0.001), the mean ± standard deviation of experiments conducted in triplicate, using one‐way ANOVA. Scale bars, 10 or 30 μm. The average fluorescence intensity per cell was analyzed using imagej (*n* = 20 cells per group). ACC1, acetyl‐CoA carboxylase 1; AKT, protein kinase B; CC, cervical cancer; Co‐IP, co‐immunoprecipitation; FASN, fatty acid synthase; mTOR, mammalian target of rapamycin; p‐AKT, phosphorylated protein kinase B; p‐mTOR, phosphorylated mammalian target of rapamycin; RACK1, receptor for activated C‐kinase 1; SREBP1, sterol regulatory element binding protein 1.

The AKT/mTOR pathway plays a central role in the regulation of cell lipid metabolism. As a multifunctional scaffold protein, RACK1 can bind to a variety of proteins in the cell, regulate protein activity, and affect the growth and differentiation of tumor cells [12]. Therefore, it was hypothesized that RACK1 may activate AKT/mTOR signaling by interacting with AKT in cervical cancer cells. To explore whether RACK1 interacts with AKT in CC cells, the interaction between RACK1 and AKT was analyzed and confirmed by Co‐IP and western blotting. These results suggested that knockdown of RACK1 significantly decreased the ability of RACK1 to bind to AKT (Fig. 4A). Western blot analysis of key AKT/mTOR signaling proteins included phospho‐AKT (ser472 + S474 + S473) (P‐Akt), total AKT (AKT), phospho‐mTOR (S2448) (p‐mTOR), and total mTOR (mTOR) at post‐transcriptional levels. Knockdown of RACK1 significantly decreased the expression levels of phosphorylated AKT and mTOR, but the total expression levels of AKT and mTOR were not significantly changed (Fig. 4B and Fig. S4A).

Next, to detect the role of AKT/mTOR signaling in RACK1‐promoted fatty acid synthesis metabolism in cervical cancer cells, we treated CC cells with SC79 (Akt activator). Western blot analysis showed that SC79 treatment significantly enhanced the expression levels of p‐Akt, p‐mTOR, and the fatty acid synthesis enzymes (ACC1 and FASN) in CC cells with RACK1 knockdown (Fig. 4C and Fig. S4B). Meanwhile, in CC cells, the intracellular free fatty acid content and neutral lipid BODIPY staining level increased after SC79 treatment, and recovered to the level of control cells (Fig. 4D,E and Fig. S4C). These results indicated that RACK1 increased fatty acid synthesis by activating the AKT/mTOR signaling pathway.

### 3.5 RACK1 increased SREBP1 mediated fatty acid synthesis by activating AKT/mTOR signaling

**Fig. 5:**
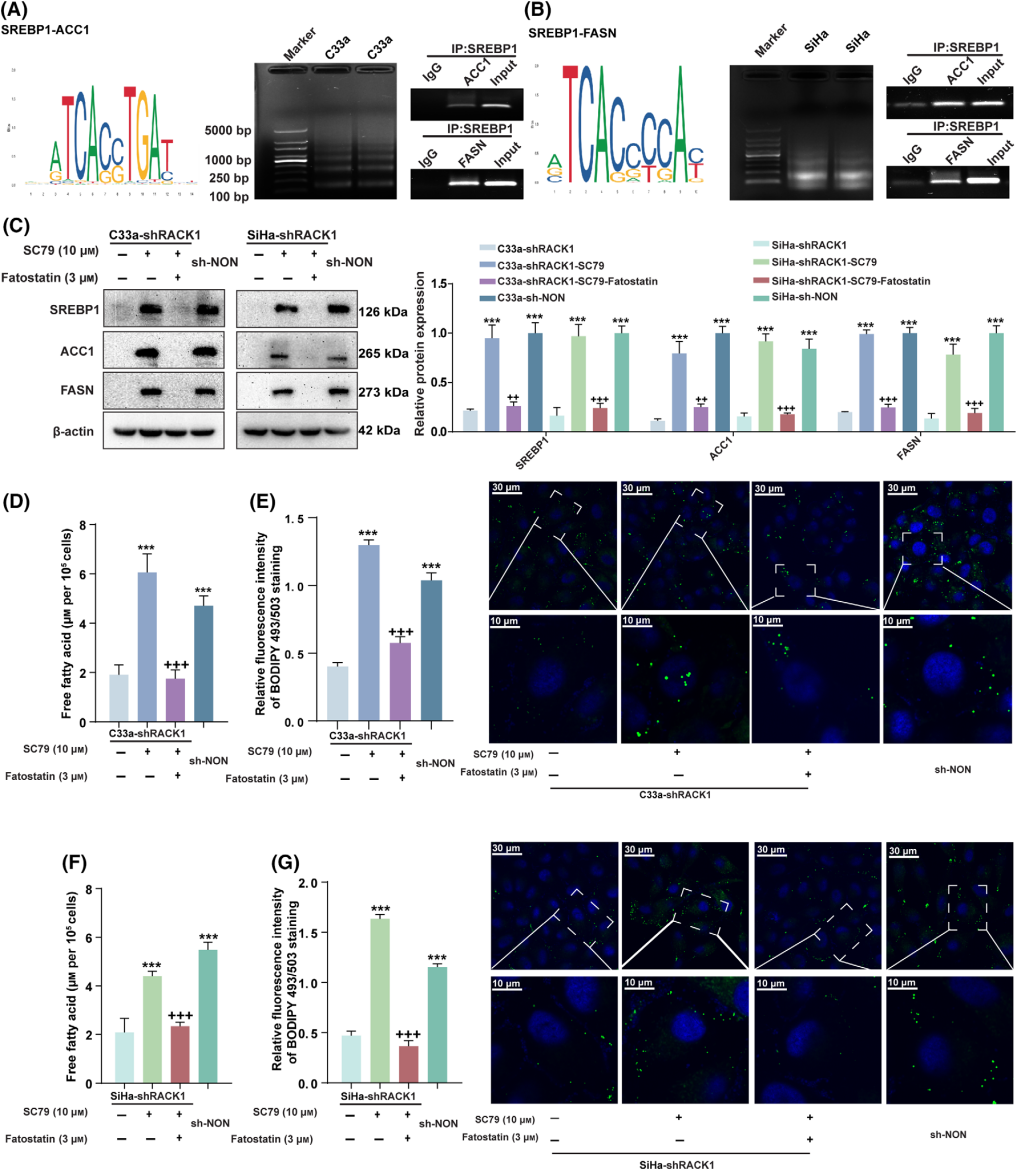
RACK1 increased fatty acid synthesis via up‐regulating SREBP1. (A, B) The JASPAR website was used to predict potential SREBP1 binding sites on the promoters of ACC1 (A, left) and FASN (B, left). The result of the target DNA fragment (A, B, middle). ChIP to identify *ACC1* (A, right) and *FASN* (B, right) as direct target genes of SREBP1 in CC cells (*n* = 3). shRACK1 cells was stimulated with SC79 and/or Fatostatin. (C) The SREBP1, ACC1 and FASN expression were examined using western blot analysis (left), with the protein bands assessed (right, *n* = 3). (D) Intracellular free fatty acid levels of C33a cell (*n* = 3). (E) Neutral lipid content detection by staining with fluorescence dye BODIPY 493/503 in C33a cell (right), with the quantified bands assessed (left, *n* = 3). (F) Intracellular free fatty acid levels of SiHa cell (*n* = 3). (G) Neutral lipid content detection by staining with fluorescence dye BODIPY 493/503 in SiHa cell (right), with the quantified bands assessed (left, *n* = 3). Data were compared with the shRACK1 group (***P < 0.001), or shRACK1‐SC79 group (^++^ *P* < 0.01 or ^+++^ *P* < 0.001), the mean ± standard deviation of experiments conducted in triplicate, using one‐way ANOVA. Scale bars, 10 or 30 μm. The average fluorescence intensity per cell was analyzed using image j (*n* = 30 cells per group). ACC1, acetyl‐CoA carboxylase 1; CC, cervical cancer; ChIP, Chromatin Immunoprecipitation; FASN, fatty acid Synthase; RACK1, Receptor for activated C‐kinase 1; SREBP1, sterol regulatory element binding protein 1.

RACK1 increased the levels of fatty acid and protein, suggesting regulation at transcriptional level. To explore the molecular mechanism by which RACK1 regulated fatty acid synthesis enzymes (ACC1, FASN), the levels of sterol regulatory elementary binding protein 1 (SREBP1), crucial transcriptional regulator in the regulation of lipogenic gene expression [13], were determined by western blot analyses in CC cells. SREBP1 significantly decreased when RACK1 was knocked down (Fig. 4B and Fig. S4A). Furthermore, to prove that ACC1 and FASN were direct targets of SREBP1 in CC cells, the JASPAR online database was used to predict potential SREBP1‐binding sites on the ACC1 and FASN promoters. ChIP assays were subsequently performed to further confirm that *ACC1* and *FASN* were direct target genes of SREBP1 in the two cervical cancer cell lines (Fig. 5A,B). Next, to investigate whether SREBP1 was involved in RACK1‐upregulated expressions of ACC1 and FASN and fatty acid synthesis in CC cells, we conducted a rescue experiment. Fatostatin, a SREBP1 inhibitor, effectively attenuated fatty acid synthesis in SC79‐induced CC cells (Fig. 5D–G). As shown by using intracellular fatty acid content and neutral lipid BODIPY staining level, fatostatin also eliminated SC79‐induced increased expression of enzymes associated with fatty acid synthesis (Fig. 5C).

**Fig. 6:**
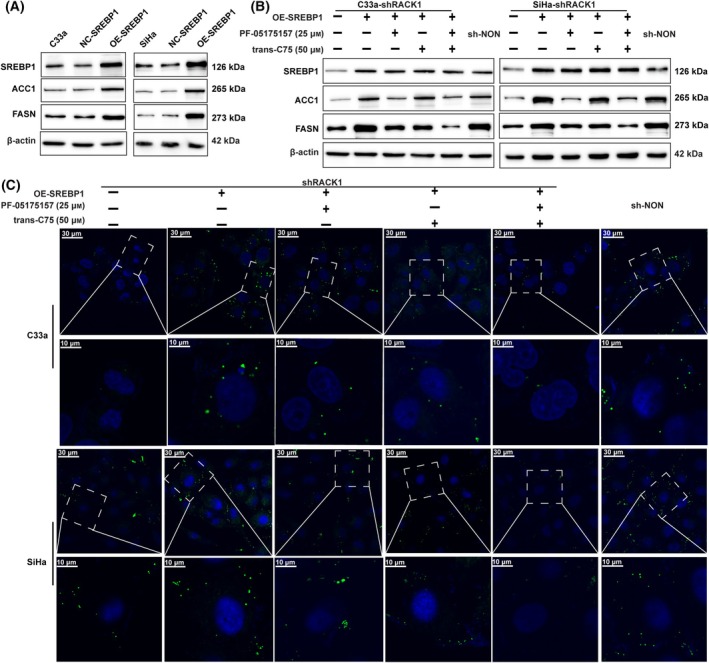
RACK1 increased SREBP1 mediated fatty acid synthesis by activating fatty acid synthesis enzymes. (A) Western blot analysis for protein expression levels of SREBP1, ACC1 and FASN in CC cell with SREBP1 overexpression (*n* = 3). The Effect of trans‐C75 and PF‐05175157on the shRACK1 cell transfected with OE‐SREBP1 lentivirus. (B) The SREBP1, ACC1 and FASN expression were examined using western blot analysis (*n* = 3). (C) Neutral lipid content detection by staining with fluorescence dye BODIPY 493/503 in CC cell (*n* = 3). Scale bars, 10 or 30 μm. ACC1, acetyl‐CoA carboxylase 1; CC, cervical cancer; FASN, fatty acid Synthase; RACK1, Receptor for activated C‐kinase 1; SREBP1, sterol regulatory element binding protein 1.

Next, to examine the involvement of FASN and ACC1 in RACK1‐promoted fatty acid synthesis, we upregulated the expression of SREBP1 in shRACK1‐CC cells through transducing lentivirusion. Then, the shRACK1‐OE SREBP1 cells treated with C75 (inhibitor of FASN) or PF‐05175157 (inhibitor of ACC). The results showed that suppression of FASN and ACC markedly attenuated SREBP1 overexpression‐increased lipid content in CC cells, as evidenced by downregulated the expression of ACC1 and FASN (Fig. 6A,B and Fig. S5C), decreased intracellular levels of free fatty acid (Fig. S5B) and reduced BODIPY staining of neutral lipids in CC cells (Fig. 6C and Fig. S5A) treated.

### 3.6 RACK1 promoted cell proliferation of CC via activating AKT/mTOR/SREBP1 pathway induced fatty acid biosynthesis enzyme

**Fig. 7:**
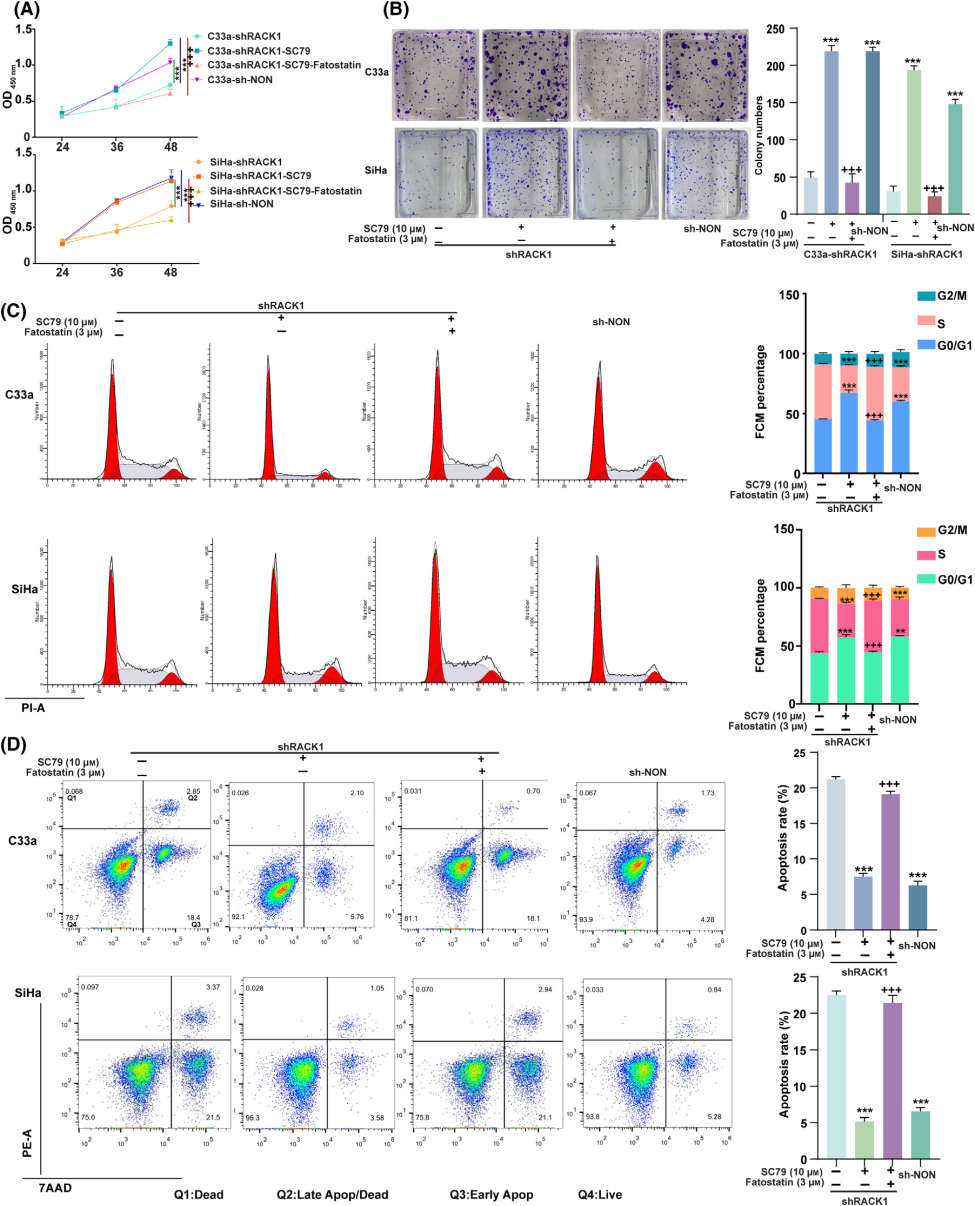
RACK1 improved cell proliferation by activating AKT/mTOR/SREBP1 pathway. shRACK1 cells was stimulated for 24 h with SC79 and Fatostatin. (A) Cell proliferation according to CCK8 assays. (B) The results of colony formation (left), with the quantified bands assessed (right). (C) The Cell cycle was examined using flow cytometry in CC cells (left), with the quantified bands assessed (right). (D) The Cell apoptosis rate was examined using flow cytometry (left), with the quantified bands assessed (right). Shown is one representative of three independent experiments. Data were compared with the shRACK1 group (**P < 0.01 or ***P < 0.001), or shRACK1‐SC79 group (^+++^ *P* < 0.001), the mean ± standard deviation of experiments conducted in triplicate, using one‐way ANOVA. AKT, protein kinase B; CC, cervical cancer; CCK8, Cell Counting Kit‐8; mTOR, mammalian target of rapamycin; RACK1, Receptor for activated C‐kinase 1; SREBP1, sterol regulatory element binding protein 1.

In this study, we aimed to determine whether the effects of RACK1 on the proliferation and apoptosis of cervical cancer cells depend on fatty acid synthesis mediated by the AKT/mTOR/SREBP1 signaling pathway. For further verification, we first treated CC cells with SC79 and fatostatin. CCK8 assay, colony formation, and flow cytometry analysis showed that SC79 treatment significantly increased the proliferation ability (Fig. 7A–C) but inhibited the occurrence of apoptosis of CC cells with RACK1 knockdown (Fig. 7D). Fatostatin also eliminated SC79‐promoted cell proliferation (Fig. 7A–C), while apoptosis was the opposite (Fig. 7D). Next, we treated shRACK1‐OE SREBP1 cells with C75 and PF‐05175157 to inhibit fatty acid synthesis‐related enzyme and then evaluated changes in cell proliferation. The results showed that suppression of fatty acid synthesis markedly attenuated growth (Figs S6A,B and S7A,C) and increased apoptosis (Figs S6C and S7B) in CC cells. These findings imply that RACK1 may play a carcinogenic role in cervical cancer by activating AKT/mTOR/SREBP1‐mediated fatty acid synthesis to promote CC cell proliferation.

## 4 Discussion

Metabolic reprogramming in tumor cells involves significant changes in cellular metabolism to support the rapid proliferation seen in tumorigenesis. To sustain the swift growth of the tumor cell population, there must be a balance between energy supply and the synthesis of biological macromolecules. This reprogramming is predominantly governed by alterations in gene expression and the metabolic network within tumor cells. It influences cellular metabolism either by directly modifying the activity of metabolic enzymes or through intricate intracellular signal transduction pathways, leading to a redirection and redistribution of nutrient flow within the metabolic network. Additionally, these metabolic changes create a conducive microenvironment that facilitates tumor cell invasion, metastasis, immune evasion, and resistance to apoptosis [14, 15].

In this study, we utilized MSI‐based spatially resolved metabolomics (SM) analysis and 10× Genomics Visium‐based spatial transcriptomics (ST) sequencing to examine cervical cancer tissues. We meticulously extracted tumor‐, epithelium‐, and stroma‐specific metabolic profiles and gene expressions for dimension reduction analysis and the identification of discriminatory variables. The combined use of SM and ST significantly enhanced our understanding of cervical cancer metabolism: While SM profiling offered a detailed overview of tumor metabolic processes, the regulation of metabolite expression by upstream genes was elucidated through ST, enabling the visualization of complex tumor metabolic reprogramming across multiple interconnected layers. Specifically, our mass spectrometry imaging focused on both cancerous and epithelial tissue sections, ensuring comprehensive visualization of metabolites. Notably, these imaged metabolites were predominantly involved in the biosynthesis of unsaturated fatty acids linked to cancer, providing a thorough exploration of the metabolic alterations associated with lipid metabolism reprogramming.

Lipid metabolism reprogramming is identified as a significant alteration in tumor cell metabolism. To meet the demands for synthesizing biological membranes and generating energy, tumor cells often enhance their lipogenesis and fatty acid (FA) oxidation processes [16, 17, 18]. In our study, we performed mass spectrometry imaging (MSI) to visualize spatial changes in 15 metabolites, including oleic acid, which is associated with lipid synthesis, in cervical cancer tissues. Some studies suggest that oleic acid induced cancer cells led to increase the expression of SREBP1, FAS, and SCD‐1 and induce tumor progression in hepatocarcinoma cells [19, 20]. Our findings indicated that oleic acid levels were elevated in the tumor regions. Subsequent ST analysis demonstrated that in Case 1, the *FASN* and *ACC1* genes, which are involved in fatty acid synthesis, were significantly upregulated in the tumor tissues. In contrast, Case 2 showed no significant differences in these analyses. This discrepancy between the two cases may be due to individual variability or a limited sample size. The spatially resolved characterization of elevated lipids and enzymes related to lipid synthesis in cervical cancer highlights the necessity for tumor cells to reconfigure their lipid synthesis and metabolic pathways. This reprogramming is essential for maintaining cellular function and activity.

In our previous research, we identified RACK1 as a potent regulator of metabolism in cervical cancer progression. RACK1 enhances glycolysis by increasing the activity of four key glycolytic enzymes, thereby promoting the glycolytic pathway [11]. In addition to its role in glycolysis, RACK1 facilitates the assembly of the Atg14L‐Beclin 1‐Vps34‐Vps15 complex, which promotes autophagy‐regulated lipid metabolism and prevents excess lipid accumulation in the liver [21]. Furthermore, Chen et al. found that RACK1 induces autophagy in macrophage‐derived foam cells by activating AMPK and interacting with ATG5, thereby preventing the buildup of lipids [22]. Despite its recognized functions, the exact role of RACK1 in lipid metabolism within cervical cancer remains unclear. Many cancers exhibit increased *de novo* fatty acid (FA) synthesis, which is believed to be a primary metabolic pathway cancer cells use for acquiring FAs [23]. Numerous enzymes associated with *de novo* FA synthesis, such as FASN, ACC, and SCD, are frequently overexpressed in various cancers, thereby boosting FA production and contributing to the malignancy of these tumors [24, 25, 26]. Furthermore, previous studies have shown that key enzymes such as ACC1 and FASN are significantly upregulated in multiple cancer types, and their elevated levels are strongly correlated with accelerated cell proliferation [27, 28]. Our spatially resolved analysis indicated that *RACK1* aligns with oleic acid and genes related to lipid synthesis within the tumor region. Based on these findings, we hypothesized that RACK1 could influence its biological functions by regulating the lipid synthesis pathway. Our results showed that RACK1 enhances *de novo* fatty acid synthesis by upregulating the expression of ACC1 and FASN, leading to lipid accumulation and promoting the proliferation of cervical cancer cells. Changes in cellular lipid content can result from alterations in lipid biosynthesis, fatty acid uptake, or fatty acid oxidation. In recent years, researchers have increasingly recognized the significance of fatty acid oxidation (FAO) in cancer metabolism, alongside lipid biogenesis, in tumorigenesis [29]. Numerous studies have demonstrated that cancer cells depend on FAO for various processes such as proliferation, metastasis, survival, and drug resistance [30]. However, our current study found no significant impact of RACK1 on fatty acid oxidation in cervical cancer cells, indicating that RACK1 may promote lipogenesis rather than lipolysis in CC.

Additionally, the Sterol Regulatory Element‐Binding Proteins (SREBPs) are a crucial family of transcription factors known to regulate lipid homeostasis [31, 32]. Two isoforms, SREBP1 and SREBP2, are expressed in mammalian cells. Elevated expression of SREBP1 has been reported in various cancers, including CC, and is known to accelerate cancer progression [33, 34, 35]. Our findings indicate that RACK1 induces the expression of SREBP1, which in turn upregulates ACC1 and FASN. SREBP1 directly binds to the promoters of *ACC1* and *FASN*, highlighting the important role of SREBP1‐mediated *de novo* lipogenesis in cancer progression. These results suggest that concurrent activation of *de novo* lipogenesis may be a common metabolic event in cancer progression, warranting further investigation.

The significance of AKT/mTOR signaling activation in cancer progression has been elucidated in numerous previous studies [11, 36], including our own. This pathway is frequently activated in various human cancers and stimulates lipid synthesis by activating SREBP1. In our current study, we demonstrated that RACK1 mediates the activation of AKT/mTOR signaling through phosphorylation, leading to increased expression of SREBP1 and its downstream targets ACC1 and FASN, thereby promoting fatty acid synthesis. Our findings suggest that the upregulation of RACK1 is common and contributes to the growth of CC cells. Since increased fatty acid levels provide essential building blocks for cancer growth, we investigated whether RACK1‐mediated lipid synthesis plays a role in CC growth. As anticipated, we observed that inhibiting FASN and ACC1, key enzymes in fatty acid synthesis, significantly reduced cell growth, highlighting the regulatory role of RACK1 in this process.

However, there are limitations to this study. First, the effects of RACK1‐mediated lipid metabolism were only explored the mechanism of fatty acid synthesis in CC cell lines, the mechanism of fatty acid oxidation and fatty acid uptake were lacking. Additionally, while we have shown that fatty acid synthesis is crucial in RACK1‐promoted CC cells, it remains unclear if there is a preferential selection order for other modes of lipid metabolism under unfavorable conditions, warranting further investigation.

Our findings suggest that RACK1 plays a role in the reprogramming of lipid metabolism in cervical cancer, ultimately influencing tumor proliferation. This suggests that RACK1 may have potential anti‐cancer effects through the regulation of cancer metabolism. However, the tumor metabolic microenvironment is governed by a complex regulatory network, and further research is needed to elucidate the precise mechanisms by which RACK1 modulates molecular pathways in lipid metabolism.

## 5 Conclusions

Our findings suggest that RACK1 plays a regulatory role in lipid metabolism by activating the AKT/mTOR/SREBP1 signaling pathway, thereby promoting fatty acid synthesis in cervical cancer cells. These results also indicate the potential of RACK1 as a valuable marker for assessing metabolic changes in cervical cancer and its implications for disease progression.

## Conflict of interest

The authors declare no conflict of interest.

## Author contributions

AH conceived the study and JM revised the manuscript. LX and JL analyzed the data and prepared the figures and tables. LX drafted the manuscript. JM collected specimens and clinical diagnosis. All authors read and approved the final manuscript.

## Supporting information


**Fig. S1.** The strategy to extract region‐specific MS spectra in heterogeneous CC tissue.
**Fig. S2.** Tumor morphology and hierarchical clustering results.
**Fig. S3.** RACK1 significantly improved lipid contents and expression levels of fatty acid in CC cells.
**Fig. S4.** Identification of signaling pathway in the RACK1 improved lipid contents of CC cells.
**Fig. S5.** RACK1 increased SREBP1‐mediated fatty acid synthesis by enhancing fatty acid synthesis enzymes.
**Fig. S6.** RACK1 improved cell proliferation by enhancing fatty acid synthesis enzymes.
**Fig. S7.** RACK1 improved cell proliferation by enhancing the expression of fatty acid synthesis enzymes.

**Table S1.** Information of primary antibodies used in the present study.

**Table S2.** Primer sequence and promoter primer sequence.

**Table S3.** Basic sequencing data of the 2 CC samples.

## Data Availability

All data generated or analyzed during this study are included in this publication and/or are available from the corresponding author upon reasonable request.
