# Supplementary material for: Combined spatially resolved metabolomics and spatial transcriptomics reveal the mechanism of RACK1‐mediated fatty acid synthesis
Source: Mol Oncol. 2024 Oct 18;19(6):1668–86. doi: 10.1002/1878-0261.13752 (PMC12161477; doi:10.1002/1878-0261.13752)
Supplement: Supplementary file 1 — Fig. S1. The strategy to extract region‐specific MS spectra in heterogeneous CC tissue. Fig. S2. Tumor morphology and hierarchical clustering results. Fig. S3. RACK1 significantly improved lipid contents and expression levels of fatty acid in CC cells. Fig. S4. Identification of signaling pathway in the RACK1 improved lipid contents of CC cells. Fig. S5. RACK1 increased SREBP1‐mediated fatty acid synthesis by enhancing fatty acid synthesis enzymes. Fig. S6. RACK1 improved cell proliferation by enhancing fatty acid synthesis enzymes. Fig. S7. RACK1 improved cell proliferation by enhancing the expression of fatty acid synthesis enzymes. [file MOL2-19-1668-s002.zip › Supporting information.docx]

Supporting information

Fig.S1. The strategy to extract region-specific MS spectra in heterogeneous CC tissue. (A) H&E-stained tissue images of case 2 with marked cancer (red), normal (blue), and stromal (green) tissue regions (left), the MSI of case 2 (middle), the microscopy- MSI overlay of case 2 (right). (B) OPLS-DA score plots for cancer tissue (red triangle), stromal tissue (green dot) and epithelial tissue (blue square) of case 2. (C) Representative mass spectra for stromal tissue (C1), normal epithelium tissue (C2) and cancerous tissue (C3). (D1-3) Digitised image of the corresponding H&E stained section for this sample. (E)Metabolic pathway analysis of case 2. (F) The IC50 values of CC cells treated with oleic acid was detected with CCK-8 assay. (G-H) The cell activity of cell line treated with different concentrations oleic acid (0μM，10μM and 25μM) was detected with (G) CCK-8 assay and (H) colony formation. Scale bars, 1000 μm. MS, Mass Spectrometry; CC, cervical cancer; H&E, Hematoxylin Eosin; MSI, Mass Spectrometry Imaging; OPLS-DA, Orthogonal Partial Least Squares Discriminant Analysis; CCK8, Cell Counting Kit-8.

Fig.S2. Tumor morphology and hierarchical clustering results. (A1) H&E-stained tissue images of case 2 with marked cancer (red, left), normal (blue, right), and stromal (green, right) tissue regions. (A2) UMAP plot performed on the gene expression data from spots covered by tissue. (A3) UMAP clustering of normal (left) and cancer (right) tissues has identified 7 groups of spots, which have been assigned a color each. (B) The spatial distributions of ACC1 gene (B1, top left and middle) and FASN gene (B2, top left and middle). UMAP plot of case 2 performed on the ACC1 (B1, top right) and FASN (B2, top right) expression data from spots covered by tissue. MS images and levels of oleic acid (B1 and B2, middle). The overlay of ACC1 spatial expression and oleic acid (B1, bottom left and middle), FASN spatial expression and oleic acid (B2, bottom left and middle). (C) Violin Plot of ACC1 (left) and FAS (right) gene expression across 4 clusters with 3569 spots in CC (cluster 4,5,7 with 3341 spots (cancer) and cluster 6 with 228 spots (normal)). Data were represented as mean±standard. and compared with the NC, (NS., no significance; independent sample t-tests were used for comparisons between two groups). Scale bars, 1000 μm. H&E, Hematoxylin Eosin; UMAP, Uniform Manifold Approximation and Projection; ACC1, acetyl-CoA carboxylase 1; FASN, fatty acid Synthase; MS, Mass Spectrometry; CC, cervical cancer; NC, normal control.

Fig.S3. RACK1 significantly improved lipid contents and expression levels of fatty acid in CC cells. (A1) UMAP plot performed on the RACK1 expression data from spots covered by cancerous (left) and normal (right) tissues of case 2. (A2) The spatial distributions of RACK1 gene. (A3) MS images and levels of oleic acid in cancerous (left) and normal (right) tissues. (A4) The overlay of RACK1 spatial expression and oleic acid in cancerous (left) and normal (right) tissues. (B) Violin Plot of RACK1 gene expression across 4 clusters with 3569 spots in CC (cluster 4,5,7 with 3341 spots (cancer) and cluster 6 with 228 spots (normal)). (C) Spatial transcriptomics images demonstrated enrichment of ACC1, RACK1, and FAS transcript levels to be co-localized in the cancer region of case 2. The spatial distributions of ACC1 (top left), RACK1 (middle), and FASN (bottom left), the overlay of ACC1 spatial expression and RACK1 spatial expression (top right), and the overlay of FASN spatial expression and RACK1 spatial expression (bottom right). (D) Western blot analysis detected protein expression of the ACC1, FASN, CD36 and CPT1A in CC cells with RACK1 knockdown. (E) The graphs represent densitometry quantification of immunoblots normalized to β-actin (n = 3). (F) Spearman correlation to analyze the correlation between the mRNA expression of RACK1 and ACC1 and FASN in tumor tissues from 25 CC patients. Data were compared with NC (***P<0.001) or shRACK1, (***P<0.001，independent sample t-tests were used for comparisons between two groups, the mean±standard deviation of experiments conducted in triplicate, using one-way ANOVA). Scale bars, 1000 μm. RACK1, Receptor for activated C-kinase 1; CC, cervical cancer; UMAP, Uniform Manifold Approximation and Projection; MS, Mass Spectrometry; ACC1, acetyl-CoA carboxylase 1; FASN, fatty acid Synthase, NC, normal control.

Fig.S4. Identification of signaling pathway in the RACK1 improved lipid contents of CC cells.

(A) The quantified bands were assessed for the expression of p-AKT (ser472 +S474 + S473), AKT, p-mTOR (S2448), total mTOR and SREBP1 (n = 3). shRACK1 cells was stimulated with SC79. (B) The quantified bands were assessed for the expression of RACK1, p-AKT (ser472 + ser474 + ser473), p-mTOR (ser2448), AKT, mTOR, SREBP1, ACC1 and FASN in C33a (up) and SiHa (down) cell (n = 3). (C) Neutral lipid content detection by staining with fluorescence dye BODIPY 493/503 in SiHa cell (left), with the quantified bands assessed (right, n = 3). Data were compared with the shRACK1 group (N.S, no significance, **P<0.01, ***P<0.001), the mean±standard deviation of experiments conducted in triplicate, using one-way ANOVA. The average ﬂuorescence intensity per cell was analyzed using image J (Scale bars, 10 μm or 30 μm. N = 30 cells per group). RACK1, Receptor for activated C-kinase 1; CC, cervical cancer; Co-IP, Co-Immunoprecipitation; AKT, protein kinase B; p-AKT, phosphorylated protein kinase B; p-mTOR, phosphorylated mammalian target of rapamycin; mTOR, mammalian target of rapamycin; SREBP1, sterol regulatory element binding protein 1; ACC1, acetyl-CoA carboxylase 1; FASN, fatty acid Synthase.

Fig.5S. RACK1 increased SREBP1 mediated fatty acid synthesis by enhancing fatty acid synthesis enzymes. The Effect of trans-C75 and PF-05175157on the shRACK1 cell transfected with OE-SREBP1 lentivirus. (A) The quantified bands were assessed for neutral lipid content (n = 3). (B) Intracellular free fatty acid levels (n = 3). (C) The quantified bands were assessed for the expression of SREBP1, ACC1 and FASN (n = 3). Data were compared with the shRACK1, (***P<0.001) or shRACK1-OE SREBP1 group (^+++^P<0.001), the mean±standard deviation of experiments conducted in triplicate, using one-way ANOVA. RACK1, Receptor for activated C-kinase 1; SREBP1, sterol regulatory element binding protein 1; ACC1, acetyl-CoA carboxylase 1; FASN, fatty acid Synthase.

Fig.6S. RACK1 improved cell proliferation by enhancing fatty acid synthesis enzymes. The Effect of trans-C75 and PF-05175157on the shRACK1 cell transfected with OE-SREBP1 lentivirus. (A) The results of colony formation. (B) The Cell cycle was examined using flow cytometry in CC cells. (D) The Cell apoptosis rate was examined using flow cytometry. The average ﬂuorescence intensity per cell was analyzed using image J (Scale bars, 10 μm. N = 30 cells per group). RACK1, Receptor for activated C-kinase 1; SREBP1, sterol regulatory element binding protein 1; CC, cervical cancer. RACK1, Receptor for activated C-kinase 1; SREBP1, sterol regulatory element binding protein 1.

Fig.S7. RACK1 improved cell proliferation by enhancing the expression of fatty acid synthesis enzymes. The Effect of trans-C75 and PF-05175157on the shRACK1 cell transfected with OE-SREBP1 lentivirus. (A) The quantified bands were assessed for neutral lipid content (n = 3). (B) The quantified bands were assessed for cell apoptosis rate (n = 3). (C) The quantified bands were assessed for cell cycle (n = 3). Data were compared with the shRACK1, (***P<0.001) or shRACK1-OE SREBP1 group (^+++^P<0.001), the mean±standard deviation of experiments conducted in triplicate, using one-way ANOVA. RACK1, Receptor for activated C-kinase 1; SREBP1, sterol regulatory element binding protein 1.
