# Supplementary figures and images for: Combined spatially resolved metabolomics and spatial transcriptomics reveal the mechanism of RACK1‐mediated fatty acid synthesis
Source: Mol Oncol. 2024 Oct 18;19(6):1668–86. doi: 10.1002/1878-0261.13752 (PMC12161477; doi:10.1002/1878-0261.13752)

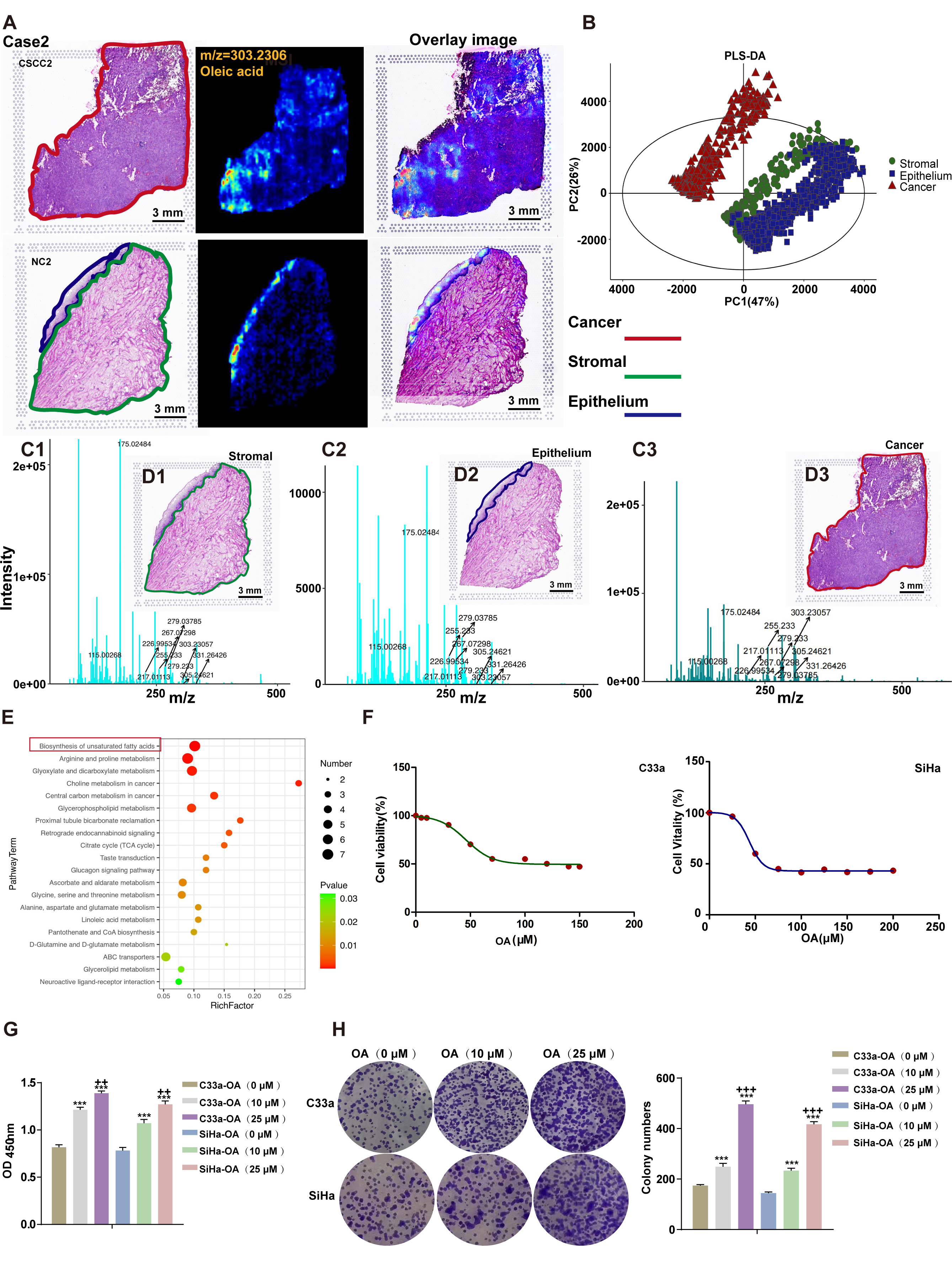

Supplement: Supplementary file 1 — Fig. S1. The strategy to extract region‐specific MS spectra in heterogeneous CC tissue. Fig. S2. Tumor morphology and hierarchical clustering results. Fig. S3. RACK1 significantly improved lipid contents and expression levels of fatty acid in CC cells. Fig. S4. Identification of signaling pathway in the RACK1 improved lipid contents of CC cells. Fig. S5. RACK1 increased SREBP1‐mediated fatty acid synthesis by enhancing fatty acid synthesis enzymes. Fig. S6. RACK1 improved cell proliferation by enhancing fatty acid synthesis enzymes. Fig. S7. RACK1 improved cell proliferation by enhancing the expression of fatty acid synthesis enzymes. [file MOL2-19-1668-s002.zip › mol213752-sup-0001-FigureS1.tif]

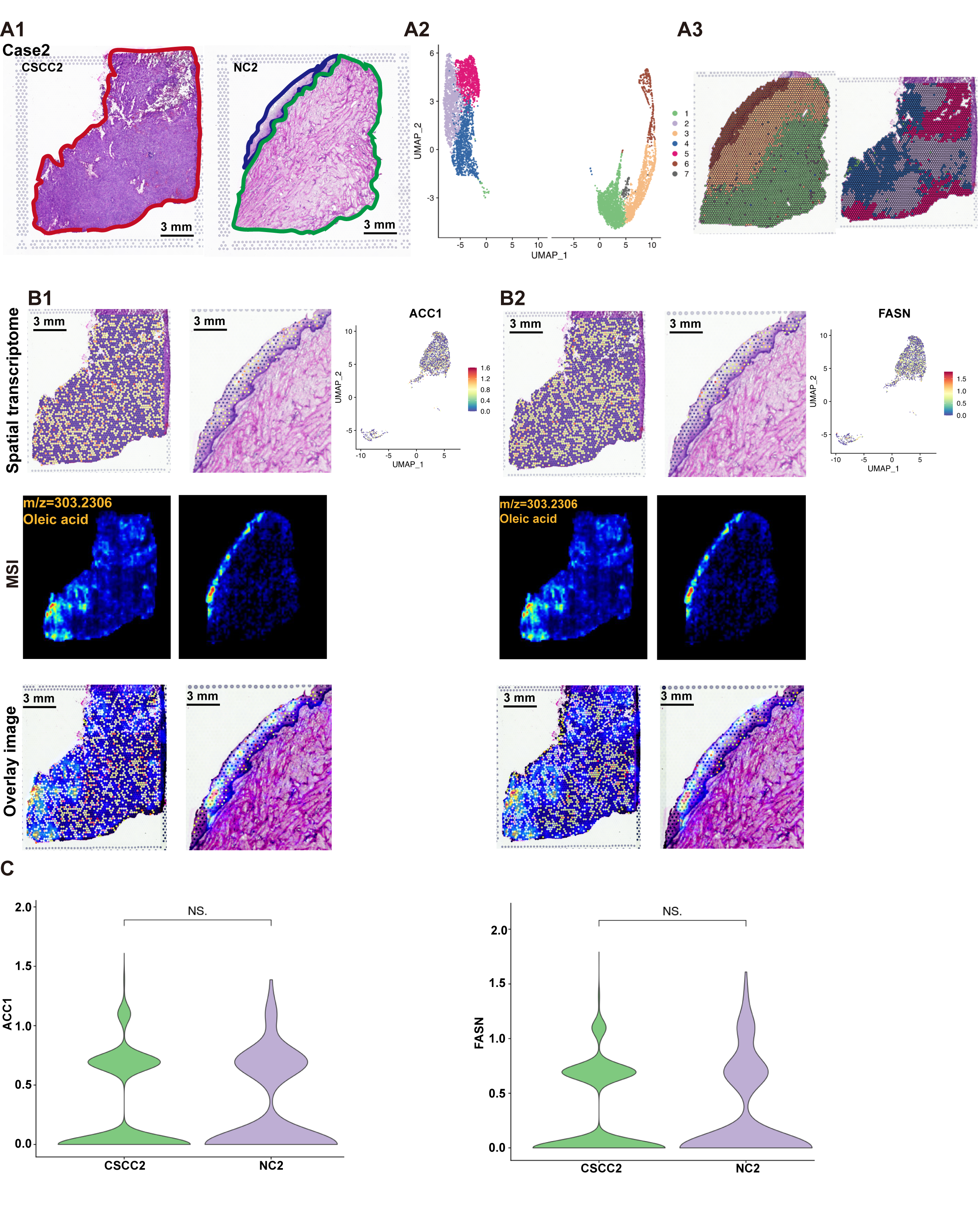

Supplement: Supplementary file 1 — Fig. S1. The strategy to extract region‐specific MS spectra in heterogeneous CC tissue. Fig. S2. Tumor morphology and hierarchical clustering results. Fig. S3. RACK1 significantly improved lipid contents and expression levels of fatty acid in CC cells. Fig. S4. Identification of signaling pathway in the RACK1 improved lipid contents of CC cells. Fig. S5. RACK1 increased SREBP1‐mediated fatty acid synthesis by enhancing fatty acid synthesis enzymes. Fig. S6. RACK1 improved cell proliferation by enhancing fatty acid synthesis enzymes. Fig. S7. RACK1 improved cell proliferation by enhancing the expression of fatty acid synthesis enzymes. [file MOL2-19-1668-s002.zip › mol213752-sup-0002-FigureS2.tif]

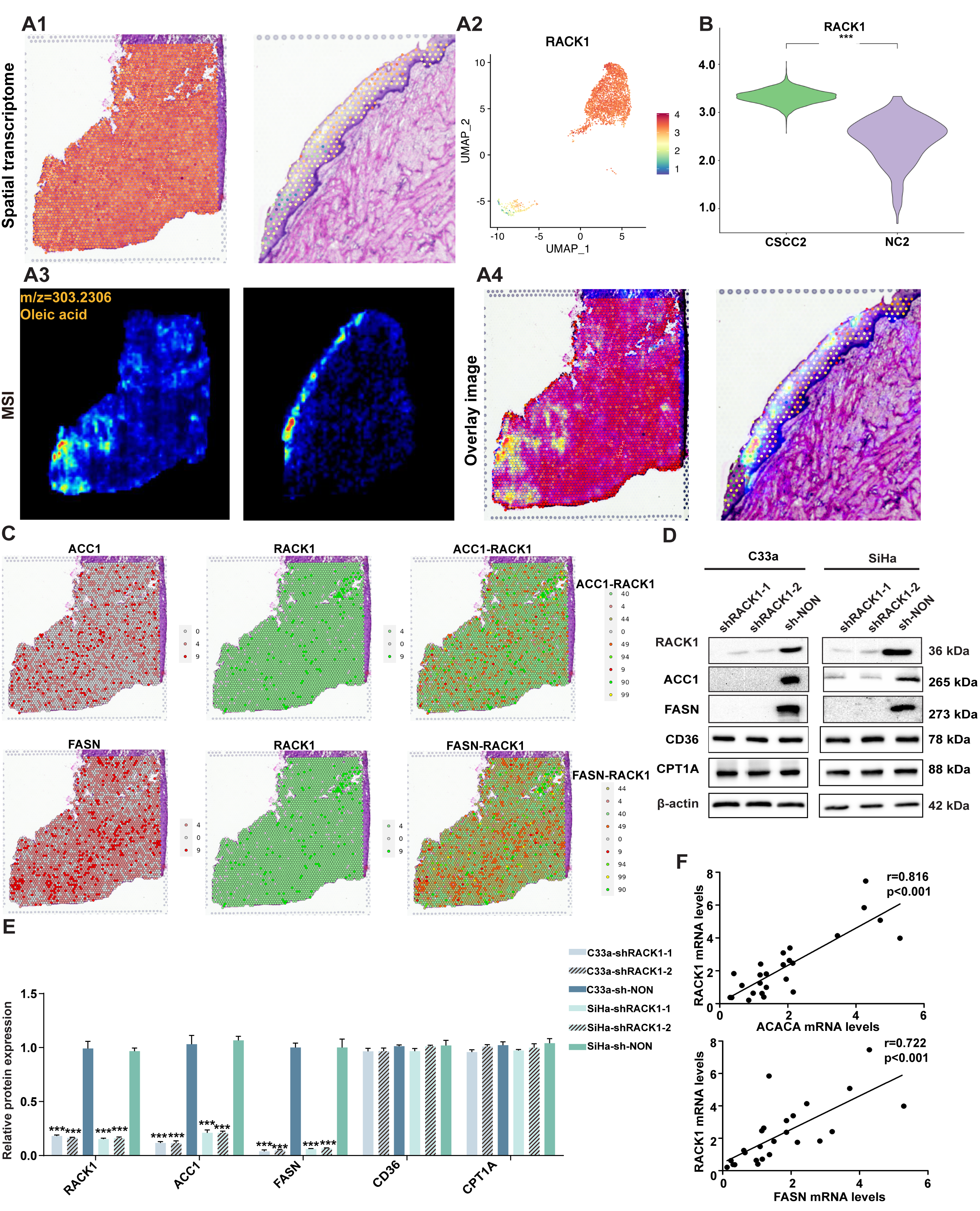

Supplement: Supplementary file 1 — Fig. S1. The strategy to extract region‐specific MS spectra in heterogeneous CC tissue. Fig. S2. Tumor morphology and hierarchical clustering results. Fig. S3. RACK1 significantly improved lipid contents and expression levels of fatty acid in CC cells. Fig. S4. Identification of signaling pathway in the RACK1 improved lipid contents of CC cells. Fig. S5. RACK1 increased SREBP1‐mediated fatty acid synthesis by enhancing fatty acid synthesis enzymes. Fig. S6. RACK1 improved cell proliferation by enhancing fatty acid synthesis enzymes. Fig. S7. RACK1 improved cell proliferation by enhancing the expression of fatty acid synthesis enzymes. [file MOL2-19-1668-s002.zip › mol213752-sup-0003-FigureS3.tif]

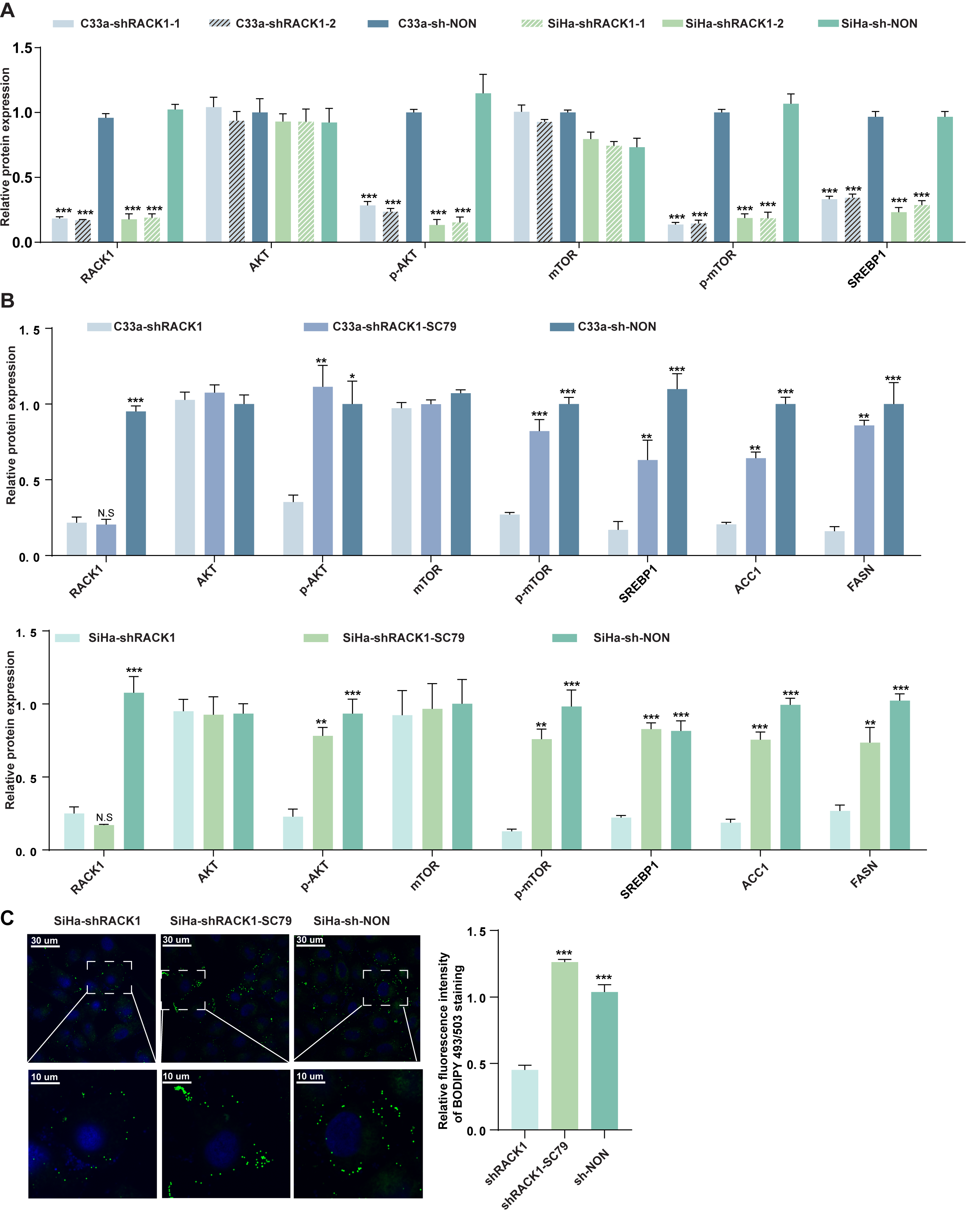

Supplement: Supplementary file 1 — Fig. S1. The strategy to extract region‐specific MS spectra in heterogeneous CC tissue. Fig. S2. Tumor morphology and hierarchical clustering results. Fig. S3. RACK1 significantly improved lipid contents and expression levels of fatty acid in CC cells. Fig. S4. Identification of signaling pathway in the RACK1 improved lipid contents of CC cells. Fig. S5. RACK1 increased SREBP1‐mediated fatty acid synthesis by enhancing fatty acid synthesis enzymes. Fig. S6. RACK1 improved cell proliferation by enhancing fatty acid synthesis enzymes. Fig. S7. RACK1 improved cell proliferation by enhancing the expression of fatty acid synthesis enzymes. [file MOL2-19-1668-s002.zip › mol213752-sup-0004-FigureS4.tif]

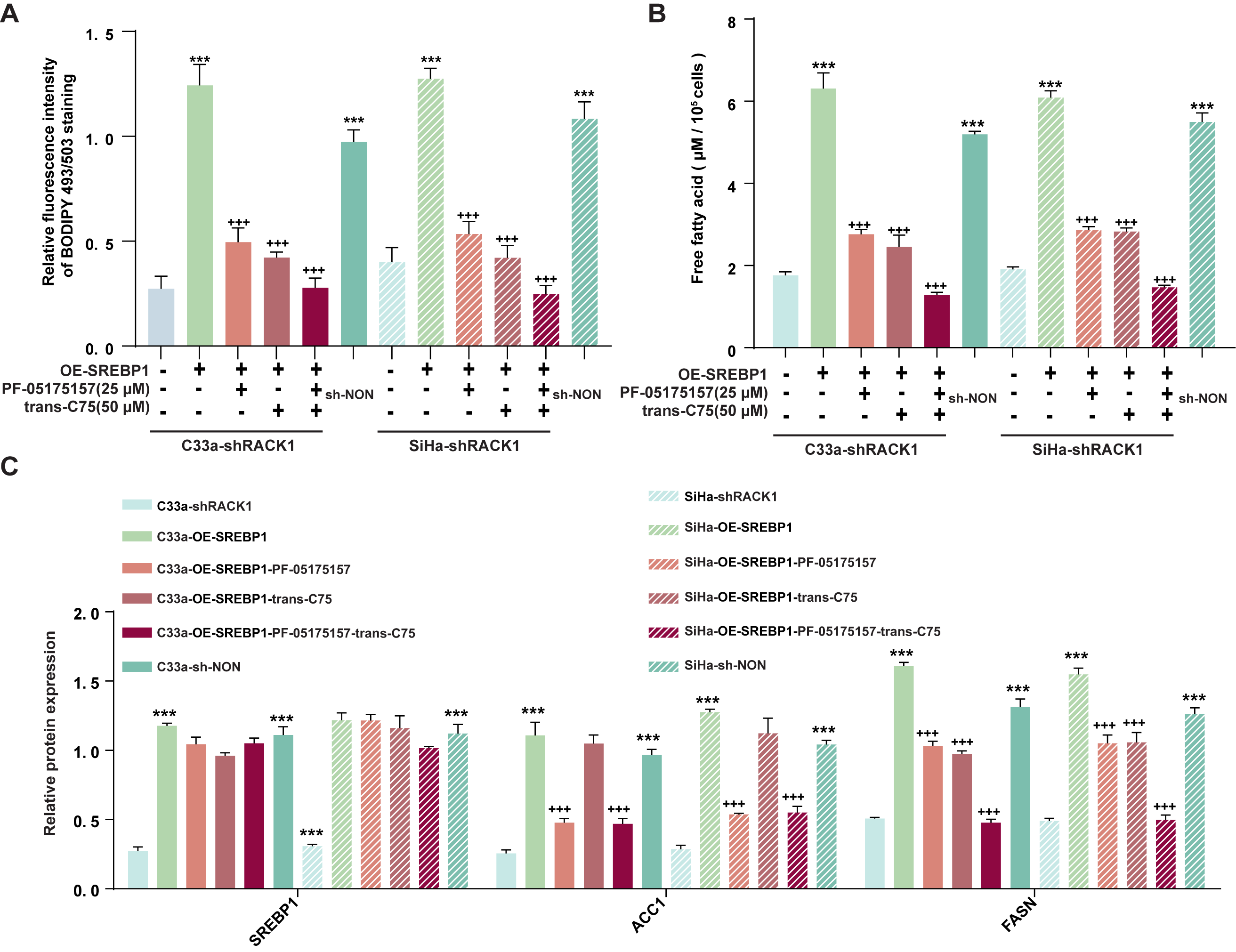

Supplement: Supplementary file 1 — Fig. S1. The strategy to extract region‐specific MS spectra in heterogeneous CC tissue. Fig. S2. Tumor morphology and hierarchical clustering results. Fig. S3. RACK1 significantly improved lipid contents and expression levels of fatty acid in CC cells. Fig. S4. Identification of signaling pathway in the RACK1 improved lipid contents of CC cells. Fig. S5. RACK1 increased SREBP1‐mediated fatty acid synthesis by enhancing fatty acid synthesis enzymes. Fig. S6. RACK1 improved cell proliferation by enhancing fatty acid synthesis enzymes. Fig. S7. RACK1 improved cell proliferation by enhancing the expression of fatty acid synthesis enzymes. [file MOL2-19-1668-s002.zip › mol213752-sup-0005-FigureS5.tif]

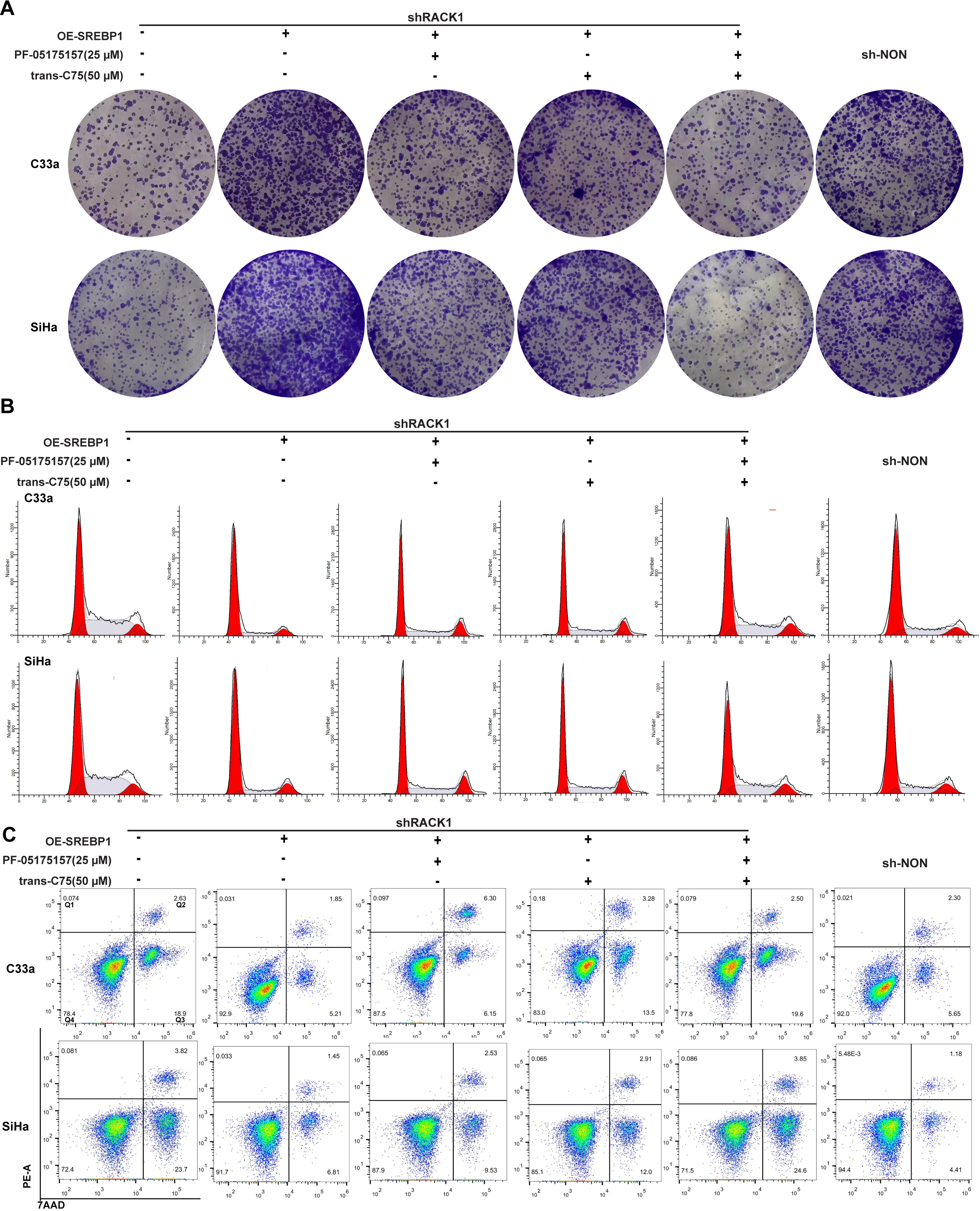

Supplement: Supplementary file 1 — Fig. S1. The strategy to extract region‐specific MS spectra in heterogeneous CC tissue. Fig. S2. Tumor morphology and hierarchical clustering results. Fig. S3. RACK1 significantly improved lipid contents and expression levels of fatty acid in CC cells. Fig. S4. Identification of signaling pathway in the RACK1 improved lipid contents of CC cells. Fig. S5. RACK1 increased SREBP1‐mediated fatty acid synthesis by enhancing fatty acid synthesis enzymes. Fig. S6. RACK1 improved cell proliferation by enhancing fatty acid synthesis enzymes. Fig. S7. RACK1 improved cell proliferation by enhancing the expression of fatty acid synthesis enzymes. [file MOL2-19-1668-s002.zip › mol213752-sup-0006-FigureS6.tif]

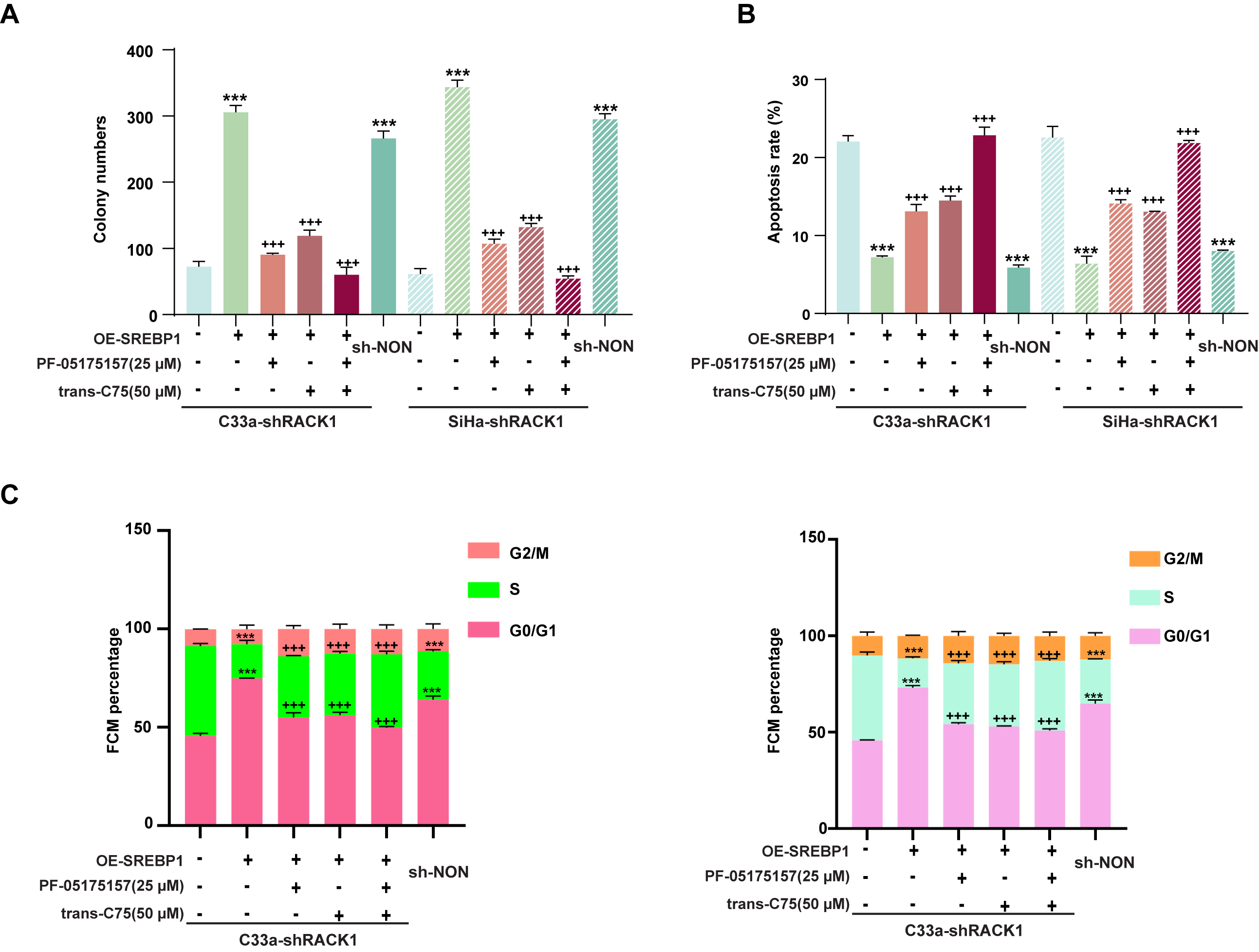

Supplement: Supplementary file 1 — Fig. S1. The strategy to extract region‐specific MS spectra in heterogeneous CC tissue. Fig. S2. Tumor morphology and hierarchical clustering results. Fig. S3. RACK1 significantly improved lipid contents and expression levels of fatty acid in CC cells. Fig. S4. Identification of signaling pathway in the RACK1 improved lipid contents of CC cells. Fig. S5. RACK1 increased SREBP1‐mediated fatty acid synthesis by enhancing fatty acid synthesis enzymes. Fig. S6. RACK1 improved cell proliferation by enhancing fatty acid synthesis enzymes. Fig. S7. RACK1 improved cell proliferation by enhancing the expression of fatty acid synthesis enzymes. [file MOL2-19-1668-s002.zip › mol213752-sup-0007-FigureS7.tif]
