# Supplementary material for: Combined spatially resolved metabolomics and spatial transcriptomics reveal the mechanism of RACK1‐mediated fatty acid synthesis
Source: Mol Oncol. 2024 Oct 18;19(6):1668–86. doi: 10.1002/1878-0261.13752 (PMC12161477; doi:10.1002/1878-0261.13752)
Supplement: Supplementary file 2 — Table S1. Information of primary antibodies used in the present study. [file MOL2-19-1668-s003.docx]

Table S1. Information of primary antibodies used in the present study

| Primary antibodies | Cat. no. | Supplier | Antibody dilution used | | | |
| --- | --- | --- | --- | --- | --- | --- |
|  |  |  | WB | CoIP | ChIP | IHC |
| Anti-human RACK1 mouse monoclonal  antibody | sc-17754 | Santa Cruz  Biotechnology, Inc. | 1:3000 | 1:50 |  | 1:200 |
| Anti-human SREBP1 rabbit polyclonal  antibody | 14088-1-AP | ProteinTech Group,  Inc. | 1:1000 |  | 1:100 |  |
| Anti-human ACC1 rabbit polyclonal  antibody | 21923-1-AP | ProteinTech Group,  Inc. | 1:5000 |  | 1:200 |  |
| Anti-Fatty Acid Synthase antibody [EPR7466] | ab128870 | Abcam | 1:10000 |  | 1:200 |  |
| Anti-human AKT rabbit polyclonal antibody | ab18785 | Abcam | 1:2000 | 1:50 |  |  |
| Anti-human pohospho-mTOR (s2448) rabbit monoclonal antibody | ab109268 | Abcam | 1:10000 |  |  |  |
| Anti-human phospho-AKT (s472 + s474 + s473) rabbit monoclonal antibody | ab192623 | Abcam | 1:2000 |  |  |  |
| Anti-human mTOR rabbit polyclonal  antibody | Ab3732 | Abcam | 1:0000 |  |  |  |
| Anti-human β-actin rabbit polyclonal  antibody | 20536-1-AP | ProteinTech Group,  Inc. | 1:8000 |  |  |  |
