# Supplementary material for: Combined spatially resolved metabolomics and spatial transcriptomics reveal the mechanism of RACK1‐mediated fatty acid synthesis
Source: Mol Oncol. 2024 Oct 18;19(6):1668–86. doi: 10.1002/1878-0261.13752 (PMC12161477; doi:10.1002/1878-0261.13752)
Supplement: Supplementary file 3 — Table S2. Primer sequence and promoter primer sequence. [file MOL2-19-1668-s004.docx]

TABLE S2 | Primer Sequence and Promoter Primer Sequence.

| Primer | Sequence |
| --- | --- |
| *RACK1* | *F: 5’-TGAGTGTGGCCTTCTCCTCT-3’*  *R: 5’-GCTTGCAGTTAGCCAGGTTC-3’* |
| *SREBP1* | *F:5’-CCAGAAACTCAAGCAGGAGAACC-3’*  *R:5’-CAGGACAGGCAGAGGAAGACG-3’* |
| *ACC1* | *F:5’-TTGCCACCCTGAGGTCTTTTT-3’*  *R:5’-TCTGAGCCAACAGAAGCAGG-3’* |
| *FASN* | *F:5’-CCCAAGGGAAGCACATT-3’*  *R:5’-GAGCGAAGTCAACACGA-3’* |
| *β-actin* | *F:5’-TAGTTGCGTTACACCCTTTCTTG-3’*  *R:5’-TCACCTTCACCGTTCCAGTTT-3’* |
| *ACC1(*Promoter ) | *F:5’-CCACACTTTTGGGGCAAGC-3’*  *R:5’-AGACACTGCGCAAGTAACATAC-3’* |
| *FASN(*Promoter ) | *F:5’-GCCCCGACGCTCATTGG-3’*  *R:5’-GCTATTTAAACCGCGGCCA-3’* |
