# Supplementary material for: Combined spatially resolved metabolomics and spatial transcriptomics reveal the mechanism of RACK1‐mediated fatty acid synthesis
Source: Mol Oncol. 2024 Oct 18;19(6):1668–86. doi: 10.1002/1878-0261.13752 (PMC12161477; doi:10.1002/1878-0261.13752)
Supplement: Supplementary file 4 — Table S3. Basic sequencing data of the 2 CC samples. [file MOL2-19-1668-s001.docx]

Table S3 Basic sequencing data of the 2 CC samples

|  | Case 1 | Case 2-CC | Case 2 1-Eepithelium and stromal |
| --- | --- | --- | --- |
| Numbers of raw reads | 343280440 | 323589818 | 286782969 |
| Numbers of genes | 24233 | 6846 | 21183 |
| Numbers of spots under tissue | 2190 | 24768 | 3341 |
| Median UMI counts per spot | 8791 | 41011 | 1670 |
| Median genes per spot | 2829 | 6846 | 839 |
| Mean reads per spot | 156749 | 96854 | 85837 |
